# Acupuncture as multi-targeted therapy for the multifactorial disease obesity: a complex neuro-endocrine-immune interplay

**DOI:** 10.3389/fendo.2023.1236370

**Published:** 2023-09-18

**Authors:** Raymond Guy Landgraaf, Michelle Nicté Bloem, Massimo Fumagalli, Marc Alexander Benninga, Fleur de Lorijn, Max Nieuwdorp

**Affiliations:** ^1^ Department of Internal and Vascular Medicine, Amsterdam University Medical Center, Amsterdam, Netherlands; ^2^ Sinomedica Gui Sheng Tang, Scientific Department, Lugano, Switzerland; ^3^ Emma Children’s Hospital, Amsterdam University Medical Center (UMC), Pediatric Gastroenterology, University of Amsterdam, Amsterdam, Netherlands; ^4^ Department of Experimental Vascular Medicine, Amsterdam University Medical Center, Amsterdam, Netherlands

**Keywords:** obesity, weight-loss, acupuncture, chronic low-grade inflammation, gut microbiota dysbiosis, appetite regulating hormones, leptin resistance, insulin resistance

## Abstract

The prevalence of obesity has reached pandemic dimensions. It is associated with multiple comorbidities and is becoming a clinical and public health threat. Obesity is a multifactorial disease with a complex pathophysiology and interplay of various systems. A strong interplay exists between the neuro-endocrine system, the immune system with systemic chronic low-grade inflammation, and microbiome dysbiosis that can lead to the development of obesity, which in turn can exacerbate each of these factors, hence creating a vicious cycle. The conventional treatment with lifestyle modifications such as diet, physical exercise, pharmacotherapy, and bariatric surgery does not always result in sufficient weight control thus paving the way for other strategies. As one such strategy, acupuncture is increasingly used worldwide to treat obesity. This narrative review outlines the evidence for this neuro-endocrine-immune interplay in the pathophysiology of obesity. Furthermore, the existing experimental and clinical evidence of acupuncture as a multi-targeted therapy for obesity is explained and future research perspectives are discussed.

## Introduction

Obesity is a complex chronic disease with a multifactorial nature which has now reached pandemic levels affecting all genders and all ages. (1–3). The prevalence of obesity worldwide has nearly tripled since 1975 (4) with more than one billion (39 million children, 340 million adolescents and 650 million adults) worldwide being obese (Body Mass Index (BMI) ≥30kg/m2) (1, 2, 5–8) and with numbers still increasing.

Obesity is caused by an imbalance in energy intake and expenditure, mainly due to a Western sedentary lifestyle and unbalanced eating patterns leading to the expansion of adipose tissue. It is often complicated by metabolic disturbances stemming from insulin resistance (IR) resulting in hyperglycemia, dyslipidemia, and hypertension, collectively referred to as metabolic syndrome (MetS) (9). In addition, several studies have shown a link between high BMI and an extensive range of non-communicable diseases (NCDs), such as cardiovascular disease, Type 2 Diabetes Mellitus (T2DM), several malignancies, musculoskeletal and rheumatological diseases, chronic kidney disease, acute respiratory infections like COVID-19 and mental disorders (1, 5). Moreover, obesity is linked to Non-Alcoholic Fatty Liver Disease (NAFLD) which was recently suggested to be replaced with the name Metabolic Dysfunction-Associated Steatotic Liver Disease (MDSLD) as the term Non-Alcoholic and Fatty were thought to be stigmatizing (10). As obesity is a gateway to many NCD’s there is an urgent need to better understand the underlying mechanisms of obesity as well as develop better multifaceted therapeutic interventions.

The origin of this multifactorial disease is much more complex than a mere result of the overconsumption of high caloric foods above individual needs, and low physical activity (11). From an evolutionary perspective different kinds of explanations have been proposed (12). One of the theories is that the origin of this pandemic stems from adaptations that were previously fundamental in human evolution but have become incompatible with modern environments. This evolution resulted in a genetic selection to predispose for fat retention, leading to excess fat storage when, in modern life, physical effort is no longer needed for survival and there is large availability of high energy foods. This in turn leads to a long-term positive energy balance (2, 12). However, not all people exposed to this modern lifestyle become obese, suggesting the existence of individual genetic mechanisms (13). This evolutionary mismatch hypothesis is still under debate (14).

In addition to the abovementioned theory, multiple contributing factors are associated with weight gain or barriers to weight loss (11, 15). Obesity originates from an interaction of these factors, including social, environmental, genetic, epigenetic, physiologic, and behavioral aspects, as well as a modern sedentary lifestyle (sedentary with unhealthy diet), and cultural, neuroendocrine, and metabolic factors (2, 11, 13).

Importantly, obesity related IR is largely reversible optimising lifestyle factors that include weight loss, improved diet, regular engagement in physical activity in order to avoid sedentariness, stress reduction and sleep sufficiency (16). Lifestyle modification (dietary changes and exercise) is the first-line choice and remains the cornerstone of obesity management (11, 17). A reduction of five to ten per cent of the bodyweight is already related to clinical metabolic improvements (13). Restoring the energy disbalance by mere reduction of caloric consumption and increased exercise is not as easy as it seems. Adherence to the dietary programme is key as weight regain once caloric restriction is stopped, known as the yo-yo-effect, is a common occurrence (18). However, as adherence is usually low, most people cannot lose weight only through increased physical activity and dietary changes (19). Pharmacotherapy is indicated for BMI over 30 kg/m^2^ or BMI over 27 kg/m ^2^combined with comorbidity, or when diet and exercise programmes have not resulted in substantial weight loss, in which case a history of failure to lose weight and sustain weight loss is a prerequisite (20). Several drugs have so far been approved and glucagon-like peptide 1 receptor agonists (GLP-1RAs) have recently become more popular because of their ability to induce weight loss as a result of appetite reduction as well as their blood sugar controlling abilities, beneficial effects on cardiovascular risk factors and reduction of cardiovascular events (21–24). Long-term safety, however, has not yet been proven and medication remains associated with reports of adverse drug reactions (25). Furthermore, pediatric use or application in case of pregnancy and lactation have not yet been approved (11). Due to their severe side effects, including cardiovascular complications, depression, suicidal thoughts, and increased risk of cancer, several weight loss medications, like fenfluramine, dexfenfluramine, sibutramine and lorcaserin were withdrawn from the market (26–28). Bariatric surgery is indicated for BMI over 40 kg/m^2^ or BMI greater than or equal to 35 kg/m ^2^ combined with comorbidity, with studies reporting reduced cardiovascular risk, reduced chronic inflammation, decreased leptin levels, changes in the gut microbiota and long-term remission of T2DM (11, 17). While nowadays being the most effective treatment for obesity with expected weight loss from five per cent up to 35 per cent, bariatric surgery is accompanied by some surgical risk. Although mortality is less than one per cent, substantial amount of early and late complications might happen in 17 per cent of cases and revision has been reported in seven per cent of the cases (29). Moreover, mineral and vitamin deficiencies are common after malabsorptive operations and require replacement therapy and monitoring for life. Gastro-esophageal reflux, dumping syndrome and weight regain are among the known reported effects (29). The search therefore continues for other innovative multi-targeted therapies for sustainable long term weight loss without side effects. Acupuncture is a popular complementary therapy with mounting clinical and experimental evidence of its effectiveness for the treatment of obesity (19, 30–33). To this end, we have provided two tables with most recent therapeutic findings of acupuncture in animal studies (**Table 1**) as well as in humans (**Table 2**). Recent evidence suggests that acupuncture exerts effect on the neuroendocrine system, modulates the immune system reducing chronic low-grade inflammation and regulates the microbiota disbalance associated with obesity (30–33, 49, 51–53). In this review, the complex pathogenesis of this multifactorial disease is explored, focusing on the relationship between neuroendocrine disbalance, chronic low-grade inflammation and the microbiome dysbiosis. Next, this review will look at the evidence of acupuncture as a new multitargeted strategy to treat this complex disease, presenting experimental as well as clinical evidence, both in animal models as in humans. Finally, future perspectives are being discussed.

**Table 1:** Acupuncture as a multitarget therapeutic solution to a multifactorial disease: animal studies.

Last name of first author/year	Animal model and sample size	Intervention: type of acupuncture	Acupuncture points	Outcome	Study limitations
Shu et al. (2020) (34)	HFDI obese rats with IR N=60	EA	ST36, CV4, CV12, ST40	Increased protein expression of hypothalamic SIRT1, which downregulated gene expression of NPY and upregulated that of POMC.	Exact mechanisms of action of EA not definitely confirmed.
Leng et al. (2018) (35)	DIO rats N=36	EA	ST25, CV12, SP6, ST36	Suppressed food intake, decreased expression levels of the NPY and AgRP, and increased PoMC expression resulting in weight loss.	No limitations reported.
Li et al. (2021) (36)	HFDI obese rats N=20	EA	ST25, CV4, ST36, SP6	Increased HDL and reduced serum CRP, TG, CHO, LDL, leptin, and prostaglandin E levels.	Exact mechanism of EA in obesity regulation remains to be further explored. Regulatory pathways of EA at different acupoints remain unclear.
Han et al. (2020) (37)	HFDI obese mice N=20	MA	ST36, KI1, CV4	Supressed intestinal lipid absorption by downregulating apolipoproteins expression in the small intestine. Reduced hyperlipidemia.	Quantities of feed and faeces were not monitored. Only lipid related indicators were analysed although metabolism of other nutrients also affects the progression of NAFLD. Only a short-term (2 weeks) effect of acupuncture was described.
Lu et al. (2019) (38)	HFDI obese mice and CD mice N=63	EA	ST36, ST44	Reduced food intake, BW, triglyceride and cholesterol levels Improved glucose tolerance Suggested association with promoted adipose tissue plasticity *via* activation of sympathetic nerves.	Limited sample size. EA could induce muscle contraction and consequently consume energy.
Tang et al. (2020) (39)	HFDI obese rats N=10	EA	ST25	Increased expression of BAT markers. Stimulated SIRT-1 protein expression and other proteins triggering the browning of WAT.	No limitations reported.
Luo et al. (2018) (40)	DIO rats N=40	EA	ST36, ST40, CV3, CV4	Increased expression of SIRT-1 and deacetylation of histones in WAT.	Energy homeostasis problems of DIO models were not resolved. Other possible mechanisms of deacetylation *via* SIRT-1 in WAT not excluded
Lu et al. (2020) (41)	HFDI obese rats N=65	EA	ST25	Inhibited SAMs and the norepinephrine transporter protein SlC6a2. Increased SNS activity and thermogenesis. Regulated immunologic balance.	No limitations reported
Jie et al. (2018) (42)	DIO rats N=30	EA	ST36	Inhibited low grade inflammation. Enhanced vagal activity. Activated α7-subtype nicotinic acetylcholine cholinergic receptors in the mesenteric WAT. Inhibited proinflammatory cytokine production.	No limitations reported
Wang et al. (2019) (43)	HFDI obese rats N=14	EA	GB26	Decreased BW, WC, and visceral adipose tissues. Reduced food intake. Enhanced insulin sensitivity, glucose homeostasis, and lipid metabolism. Modified gut microbiota composition (decreased Firmicutes/Bacteroidetes ratio and increased Prevotella_9 abundance).	No limitations reported
Wang et al. (2022) (44)	HFDI obese mice N=32	EA	ST36, CV12	Reduced expression of diabetes-related markers. Increased abundance of Firmicutes. Increased Firmicutes to Bacteroidetes ratio. Decreased abundance of Bacteroidetes and Eubacterium. Downregulated serum LPS and TNF-α.	No limitations reported
Dou et al. (2020). (45)	HFDI obese mice N=30	EA	ST25, CV4, ST36, and SP6	Promoted diversity of intestinal microbiota. Possibly regulated ENS function.	To be elucidated whether and how the diversity and function of the gut flora affected the ENS function and to what extent EA promoted the recovery of the gut flora.
Si et al. (2018) (46)	HFDI obese mice N=30	EA	ST25, CV4, ST36, and SP6	Altered bacterial diversity and metabolic genes.	Short of sample.

2hPG: 2 hour postprandial blood glucose; AE: adverse events; BAT: brown adipose tissue; BW: body weight; CD: Common diet; CG: Control group; DIO: Diet-induced-obese; EA: Electro acupuncture; ENS: Enteric Nervous System; FBG: fasting blood glucose; FINS: fasting insulin; HDL-c: Highdensity lipoprotein cholesterol; HFDI: High-fat-diet-induced ; Homa-IR: homeostasis model assessment of insulin resistance ; IR: Insulin resistance; ISI: insulin sensitivity index; LDL: low-density lipoprotein; LPS: Lipopolysaccharide; MA: manual acupuncture; SAE: serious adverse events; SR + MA: systematic review and meta-analysis; TC: total cholesterol; TG: triglyceride; TNF-α: Tumor Necrosis Factor α; VLCD: very low carbohydrate diet; WAT: white adipose tissue; WC: Waist circumference; WHtR: waist-height ratio; WHR: waist to hip ratio.

**Table 2:** Acupuncture as a multitarget therapeutic solution to a multifactorial disease: human studies.

Last name of first author/year	Study subjects, amount studies & sample size	Intervention	Acupuncture points	Outcome	Adverse events	Study limitations
Wu et al. (2019) (47)	SR + MA 20 RCTs N=1,639 Patients with IR related disorders	Acupuncture, EA, sham acupuncture, Western medical treatments, medication, and lifestyle modification.	Not reported.	Decreased Homa-IR, FBG, 2hPG and FINS. Increased ISI.	No SAEs. Few mild and transient AE: digestive tract reactions, mild pain, or subcutaneous haematoma.	Small sample size, unsatisfactory quality of included RCT’s, high heterogeneity and publication bias.
Chen et al. (2020) (48)	SR + MA 33 RCTs N=2503 Overweight and obese patients.	EA, MA, acupressure, laser acupuncture, moxibustion.	ST25, SP6 and ear Shenmen and ear Stomach.	Small reduction of BW, BMI and WC. Reduced serum lipid parameters.	Seven RCTs reported AE: mild ecchymosis, abdominal discomfort, bleeding, mild tenderness, minor inflammation.	Risk of bias, imprecision, inconsistency, publication bias, moderate to low quality of evidence.
Sheng et al. (2021) (49)	RCT N=73 Perimenopausal patients with abdominal obesity.	EA and diet intervention.	Main: CV12, CV6, CV4, ST25, SP15, SP6, BL25, BL23, BL20 and other acupoints combinations.	Decreased WC, WHtR, WHR, TG, LDL levels. Decreased microbiota species abundance. Increased species diversity. Increased proportions of Klebsiella and Kosakonia.	No AEs reported.	No limitations reported.
Fumagalli et al. (2023) (50)	Retrospective chart review N=11.233. Patients with overweight and obesity.	MA combined with VLCD.	CV17, CV12, CV9, CV4, ST40, SP6 and SP9, HT7, Sishencong.	Mean weight reduction 17.5 kg after 7 months. Maximum weight reduction 29.8 ± 12 kg for obese patients (BMI ≥ 35 kg/m2).	No AEs reported.	Lacks control group and not prospective. Primary outcome only weight and BMI-loss but lacking biochemical parameters.

2hPG: 2 hour postprandial blood glucose; AE: adverse events; BAT: brown adipose tissue; BW: body weight; CD: Common diet; CG: Control group; DIO: Diet-induced-obese; EA: Electro acupuncture; ENS: Enteric Nervous System; FBG: fasting blood glucose; FINS: fasting insulin; HDL-c: Highdensity lipoprotein cholesterol; HFDI: High-fat-diet-induced; Homa-IR: homeostasis model assessment of insulin resistance; IR: Insulin resistance; ISI: insulin sensitivity index; LDL: low-density lipoprotein; LPS: Lipopolysaccharide; MA: manual acupuncture; SAE: serious adverse events; SR + MA: systematic review and meta-analysis; TC: total cholesterol; TG: triglyceride; TNF-α: Tumor Necrosis Factor α; VLCD: very low carbohydrate diet; WAT: white adipose tissue; WC: Waist circumference; WHtR: waist-height ratio; WHR: waist to hip ratio.

## Multifactorial pathophysiology of obesity

### Neuroendocrine hormonal crosstalk and pathogenesis of obesity

**Figure 1:**
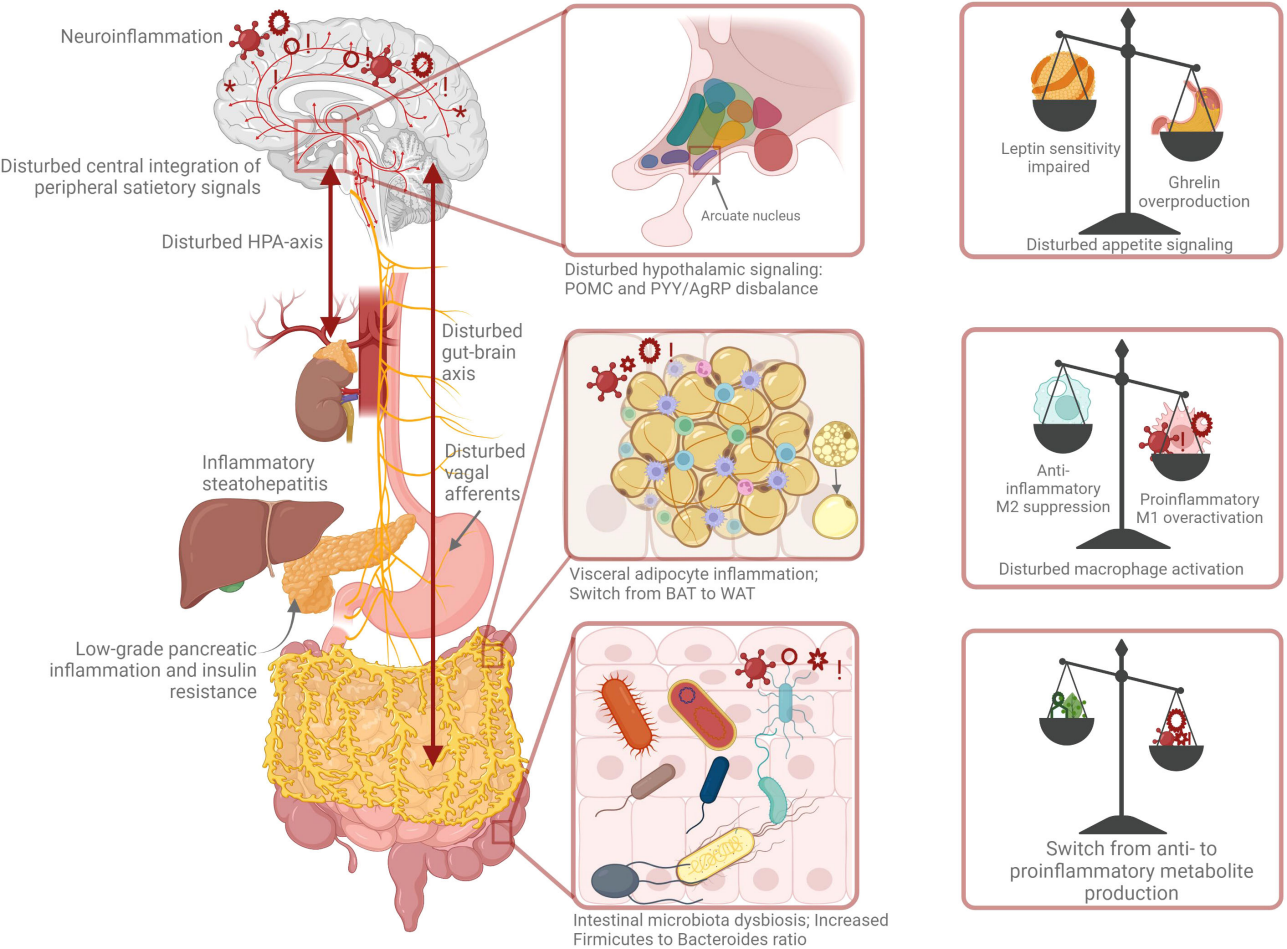
Pathophysiology of obesity: a complex orchestra of neuroendocrine cross-talk, chronic low-grade inflammation and microbiota dysbiosis.

The control of body weight through regulation of energy balance relies on a complex neuroendocrine interplay of integrating peripheral metabolic signals by the central nervous system (CNS) controlling feeding behaviour. Energy balance and food intake are controlled through a coordinated feedback system of neuropeptides and neurotransmitters in the CNS and peripheral signals arising from the gastrointestinal cells in the stomach, adipose tissue, pancreas, intestine as well as nutrients and microbiome (11, 13, 54). See **Figure 1** for this dynamic interplay in the pathogenesis of obesity.

#### Hypothalamus

The neural network in the hypothalamus regulates the energy metabolism and modulates the food intake. It is the key CNS region involved in metabolic homeostasis and consists of several interconnected nuclei responding to fluctuations in energy status through a negative feedback mechanism. These neurons form an orchestrated circuit also integrating other metabolic signals to control energy intake and expenditure (55–57). In particular, the arcuate nucleus (ARC) is of pivotal importance for primarily perceiving peripheral adiposity signals, such as leptin and insulin, and circulating nutrients, such as glucose (58). The ARC contains two populations of neurons, the first of which expresses the anorexigenic peptide proopiomelanocortin (POMC) and the second expresses the orexigenic peptide neuropeptide Y (NPY) and agouti-related protein (AgRP). They have an opposing influence on food intake. Neurons in the ARC innervate several other hypothalamic nuclei to integrate emotional and stress responses in the paraventricular nucleus (PVN). They also control the metabolic rate through the release of corticotropin-releasing hormone (CRH) and of thyrotropin-releasing hormone (TRH) (59). It has recently been found that SIRT1 or Sirtuin1 (silent mating type information regulation 2 homolog 1) plays an important role in obesity. Among other mechanisms, it regulates adiponectin secretion and inflammatory responses, and contributes to the development of IR and may be a target for IR related metabolic diseases like obesity (60). In obesity there is a hypothalamic dysregulation and disturbed appetite signaling due to a cascade of adipocyte hypertrophy and subsequent chronic low-grade inflammation, which will be further explained below.

#### Appetite-regulating hormones: leptin, ghrelin, and leptin resistance

There is a complex network of hormones and signaling molecules known as adipokines, playing a role in energy homeostasis. Leptin, resistin and adiponectin are produced by adipocytes, while the other hormones are derived from the gastrointestinal tract. Ghrelin is predominately produced by the stomach, peptide YY (PYY 3-36) and glucagon like peptide 1 (GLP-1 derive from the small intestine, and insulin and glucagon from the pancreas. Leptin and ghrelin are crucial for the neuroendocrine control of energy homeostasis. The orexigenic peptide ghrelin, also known as “the hunger hormone”, stimulates energy intake and decreases expenditure, whereas anorexigenic adipokine leptin, “the satiety hormone”, reduces energy intake and stimulates expenditure (61). The amount of leptin, produced and secreted by the adipose tissue, is in proportion to the adipose mass. Leptin participates in body weight and energy homeostasis and the impaired leptin synthesis, or sensitivity leads to disturbed energy homeostasis and body compositions (55, 62). In most cases, obese individuals have high levels of circulating leptin failing to control body weight. This suggests hypothalamic resistance to the anorectic effect of leptin (55, 63–65). The lack of response to leptin, known as leptin resistance, is a phenomenon similar to IR in patients with T2DM (66). Western lifestyle characterised by high fat and high caloric intake triggers central and peripheral leptin resistance. POMC and NPY neurons in the ARC are sites of leptin receptor expression. Leptin resistance in the ARC of the hypothalamus has been reported in obese individuals as a result of impairments at several levels in the leptin signaling pathway. Leptin resistance affects food intake regulation and insulin sensitivity, and leads to energy balance dysregulation and a subsequent vicious cycle of further weight gain and hormonal imbalance (62). This dysregulation in hypothalamic leptin signaling subsequently leads to overconsumption of nutrients and increased total body mass, thus aggravating obesity. In conclusion, a crucial hallmark for obesity is the development of resistance to leptin. Finally, ghrelin is a brain-gut peptide produced by the enteroendocrine cells in the stomach stimulating growth hormone production. Ghrelin increases in response to fasting and low nutrient availability and decreases post-prandially and prepares the body to metabolise incoming food and store energy (66). During negative energy balance, ghrelin is metabolically more active. It stimulates the AgRP and NPY expression and inhibits the POMC expression in the hypothalamus. Ghrelin blocks leptin-induced feeding reduction resulting in increased food intake and subsequent body weight (54, 67–69). In obese individuals, ghrelin levels are dysregulated, and normal function is impaired, with ghrelin resistance suggested as an underlying mechanism. However, current knowledge about ghrelin resistance is still limited and more research is needed to fully unravel its role and relevance in obesity (66).

#### Insulin and insulin resistance

Obesity is a predisposing factor for the development of T2DM as well as IR and is interconnected through numerous complex pathogenic pathways (16, 70). IR induced by obesity is characterised by impaired insulin function, as the adipose tissue induces systemic IR. IR is a key mechanism in many metabolic illnesses and not only a symptom of MetS, obesity, and hyperlipidemia, but also a premonition to T2DM, cardiovascular disease, and some cancers. There is a strong relationship between IR, visceral obesity, and increased free fatty acids (31, 71). In addition, there is a bi-directional effect of IR with the CNS where IR can be caused by a sympathetic activation. However, in turn, the related hyperinsulinemia can further activate the sympathetic branch of the autonomic nervous system (72). Furthermore, obesity is correlated to IR due to its chronic inflammatory responses. The production and secretion of proinflammatory factors like C-reactive protein, IL-6 and TNF-α are increased. This is further explained below. Next, elevated levels of lipid intermediates, free fatty acids and inflammatory cytokines in non-adipose tissues contribute to diminished insulin signalling and the insulin-resistant state present in obese individuals (13). Finally, there is a causal link between gut microbiota dysbiosis and IR (73). In overweight and obese individuals, weight loss and reduction of visceral adipose tissue improves insulin sensitivity (74).

#### Adipose tissue and lipid metabolism

Adipose tissue is a very complex endocrine organ and produces hormones such as leptin, resistin, estrogen and cytokines (75). In mammals there are three kinds of adipose tissues: white, brown, and beige. White adipose tissue (WAT) is mainly storage of energy whereas brown adipose tissue (BAT) is the main tissue to generate heat and maintain the body temperature. Beige adipose tissue also releases energy by burning calories like BAT but is genetically different. In the context of obesity, adipose tissue inflammation and altered adipokine secretion together with the deterioration of the metabolic function will be further explored below (76). In conclusion, the development of obesity is a complex interplay between leptin, the autonomic nervous system and CNS, adipose tissue, and insulin-resistance (72).

#### HPA-axis, cortisol: chronic stress & impaired sleep

Stress and obesity are closely related, with one leading to the other and vice versa (77). Stress leads to increased appetite and craving for high calorie foods, resulting in further weight gain, hence creating a vicious cycle. The stress response is regulated by the autonomic nervous system and the sympatho-adrenal-medullary system. The hypothalamus-pituitary-adrenal axis (HPA-axis) is the main regulator of the body’s stress response. The HPA-axis is triggered by several stimuli (physiological, immunological, and psychological) (77, 78). To obtain physiological homeostasis, the HPA-axis promotes the synthesis of the stress hormone cortisol, the most important glucocorticoid, amongst other hormones. Its activation starts in the hypothalamus with the release of corticotrophin-releasing hormone (CRH), stimulating the secretion of the pituitary adrenocorticotropic hormone (ACTH) which in turn stimulates the adrenal cortex to secrete cortisol (78). Murine models show significant increases in plasma ghrelin after chronic stress in rats, suggesting that appetite increases as a result of stress (77). Chronic overactivity of the HPA-axis with chronic exposure to the stress hormone cortisol has been associated with IR, as well as inflammation and redistribution of white adipose tissue to the abdominal region and can be an important contributing factor in the complex pathogenesis of obesity (78–82). Recent hair cortisol analyses show a positive association between cortisol and obesity, suggesting an altered HPA-axis setpoint and clearly demonstrating elevated serum cortisol in obese individuals (15, 81, 83, 84). Furthermore, chronic stress affects our sleeping behaviour and sleep cycle, and sleep deprivation further lead to stress, again leading to a vicious cycle. Short sleep duration is associated with increased ghrelin levels (85), leading to increased appetite and craving for high caloric foods, and is correlated with an increased risk of obesity (86). In turn, obesity can cause sleep disorders like obstructive sleep apnea, further impairing sleep quality (86, 87). Additionally, there is a bidirectional link between anxiety, depression, and obesity (88, 89). All in all, these data suggest that chronic stress is interrelated with IR, inflammation, anxiety, depression, and disturbed sleep and could lead to increased weight and obesity. See **Figure 1** for the abovementioned neuroendocrine relations.

### Chronic low grade systemic inflammation of the adipose tissue and brain

A state of chronic low-grade systemic inflammation and immune dysfunction is involved in the pathogenesis of obesity and obesity-related metabolic disorders, like MetS and T2DM (90). Adipose tissue is not only a fat storage organ, but also an important neuroendocrine organ; besides energy storage and expenditure it performs essential functions in metabolic regulation (91). In obesity, adipocyte hypertrophy, hypoxia, mechanical stress, adipocyte apoptosis, and oxidative stress together initiate an inflammatory response with infiltration of immune cells like macrophages (2). This infiltration is associated with a cell population shift from anti-inflammatory M2 macrophages to pro-inflammatory M1 macrophages, and expression of pro-inflammatory cytokines. These cytokines like Tumor Necrosis Factor α (TNFα) and Interleukin 6 (IL-6) and adipokines (leptin and resistin) then spill over into the circulation and contribute to systematic low grade chronic inflammation and dysfunction of the adipose tissue (2, 92). Pro-inflammatory adipokines secretion and decreased release of anti-inflammatory adipokines contribute to obesity-induced inflammation and promotes systemic inflammation and IR (91–93). IL-6 has a controversial role as its action varies in different cells and tissues. It can both exert a proinflammatory as well as an anti-inflammatory action (94). An acute increase due to fasting or exercise mobilizes lipids, but in contrary to this physiological elevated IL-6 level a chronic increase triggers proinflammatory action and leads to pathological conditions like dysregulation of the glucose metabolism, hepatic steatosis and insulin resistance (94).

In addition to the pro-inflammatory milieu in the adipose tissue, research has also revealed obesity related inflammatory changes perturbing brain function, especially affecting brain areas like the hypothalamus-controlling neuroendocrine functions that integrate and regulate energy homeostasis and systemic metabolism. Adipocyte hypertrophy and hyperplasia induce a state of systemic chronic low-grade systematic inflammation resulting in activation of brain immune cells such as astrocytes, microglia, and oligodendroglia. This neuroinflammation further facilitates weight gain and obesity-associated IR (95, 96). Quantitative magnetic resonance imaging (qMRI), aimed at quantifying brain water content, has recently emerged as a tool to characterise the pathophysiological process of inflammation in the brain. The hypothalamus displays the most prominent alterations in water content, but other surrounding brain regions also show signs of brain inflammation (95–97). This neuroimmune cross-talk is crucial for the understanding and pathogenesis of obesity and developing innovative therapeutic strategies. In conclusion, the intersection between the nervous system, immune system and metabolism is an important factor in the context of obesity and has recently been defined as neuro-immuno-metabolism. As mentioned earlier, in obesity a complex interactive multi-directional relationship exists between the adipose tissue, the sympathetic nervous system (SNS) and innate immune cells (72, 98), as shown in **Figure 1**. The scheme is completed by a missing link: the gut microbiota. Below, the potential causal effect of a gut microbiota disbalance on the pathogenesis of human obesity is explained.

### Gut microbiota dysbiosis and gut-brain axis

Recent research has revealed the role of the host gut microbiome in the development of obesity and its influence on host metabolism (99). The gut microbiome consists of micro-organisms present in the human gastrointestinal tract which have co-evolved into a complex system, contributing to the health status of their host (54, 100, 101). The gut microbiota comprise of a multitude of bacterial taxa, of which Firmicutes and Bacteroidetes are the most abundant phyla, accounting for over 90 per cent of the bacterial community in the large intestine (102). Normal gut microbiota exert several functions including protection against pathogens, increasing dietary energy harvest through their metabolism of carbohydrates and lipids, as well as directing energy utilisation through generation of metabolic substrates accessible to different tissues (103). Moreover, the gut microbiota affect the immune system and play an essential role in regulating the structural integrity of the gut mucosal barrier (104). Dysbiosis, described as an imbalance of microbial populations, is implicated in the pathogenesis of a large range of diseases such as inflammatory bowel disease, irritable bowel disease, neurological disorders, and cancer (105). Diet induced disruptions of the gut microbiome promote white adipose tissue inflammation and expansion, and this microbiome dysbiosis is critical in developing T2DM, MetS and obesity (105).

Indeed, in addition to its well-described contribution to digestion of food, gut microbiota composition is thought to critically influence host health and disease in the context of the gut brain axis (106, 107), where dysbiosis with concomitant increase in pathogens can lead to inflammation and disease (88, 108, 109). The regulatory system linking the CNS and enteric nervous system (ENS) to the peripheral intestinal functions is referred to as the “gut brain axis”. This complex dynamic bidirectional system includes the parasympathetic and SNS (110, 111), immune and endocrine factors as well as the gut microbiota, all regulating gastrointestinal homeostasis (110–114). The vagal nerve, as main component of the parasympathetic nervous system, is important in the regulation of the gastrointestinal system (112), exerting both excitatory as well as inhibitory control over the gastrointestinal tract (115). The role of the SNS is mainly inhibitory (111) and it is likely that its activation *via* release of stress-hormones such as epinephrine, norepinephrine and cortisol, contributes to a dysregulation of gut motility (54, 116).

In the context of metabolic diseases such as obesity, the gut brain axis integrates cerebral and gastrointestinal functions in a bidirectional manner, including gut motility, appetite, and body weight control (108). From the gut to the brain, the secretion of metabolites by the gut microbiota promotes the release of intestinal peptide hormones and neurotransmitters, such as PYY, GLP-1, 5-hydroxytryptamine (5-HT) and gamma-aminobutyric acid (GABA) (117–119). In addition to contributing to regulating peripheral metabolic status (108), these metabolites are thought to influence the CNS *via* the ENS (120). *Via* the hypothalamic ARC, they can control the balance of appetite (108, 119, 121, 122). Although the role of 5-HT in obesity has not yet been fully clarified, changes in central and peripheral levels of 5-HT have been implicated in obesity as mediators of feeding behavior and feeling of satiety (119, 122–124).

Pathways in the gut brain axis include alterations in secretion of gastrointestinal fluids, gut motility, modulating gut permeability and mucosal immune response (111). The gut microbiota is affected both directly and indirectly by neuroendocrine efferent systems comprising of the CNS and the HPA-axis (111, 121). In the context of metabolic disorders, the nervous system and HPA axis are thought to influence the gut microbiota composition, specifically the Firmicutes-to-Bacteroidetes ratio, which is of potential relevance in the development of obesity (88, 125). Initial key research in obese mice has shown a lower gut microbiota diversity and an increased Firmicutes-to-Bacteroidetes ratio compared to lean counterparts (126), and germ-free mice colonised with obesity-associated gut microbiota developed an associated phenotype (125). In human adult twins, obesity was associated with a lower Bacteroidetes abundance, and fecal transplantation thereof into mice revealed further transmissible modifying effects of human gut microbiota on obesity phenotype (127). However, although promising, this microbiota profile does not translate as such in clinical studies in adults with obesity (128). A systematic review of six placebo controlled RCTs reported that fecal microbiota transplantations (FMT) from lean donors into adults with obesity show no improvement of parameters including hepatic insulin sensitivity, BMI, fasting plasma glucose, or cholesterol level, other than a reduction of HbA1c levels at six weeks post-FMT (129). Thus, the regulation of energy metabolism of the host is influenced by many metabolites produced and modified by the gut microbiota as part of the gut brain axis (128). However, this complex interaction is not yet fully explained. Moreover, inherent to the complexity of this bi-directional system, it is likely only one of several ways in which gut microbiota contribute to host health in the context of the gut brain axis. Nevertheless, demonstrating whether specific gut microbiota-based interventions have beneficial effects on weight management and subsequently improved cardiometabolic risk remains a challenge (109, 128). Thus, other interventions should be taken into account.

## Acupuncture as a multitarget therapeutic solution to a multifactorial disease

The use of acupuncture in treating overweight and obesity has been a subject of research over the last few decades and has emerged as a safe and effective complementary treatment of obesity (31, 33, 48, 130–132). Acupuncture, used for over 3,000 years to treat various diseases, is performed by inserting fine needles through the skin into specific body sites, called acupoints. Because of its clinical efficacy, cost efficiency and limited side effects, the use of acupuncture is recommended for a wide variety of diseases by the WHO (133, 134). It is based on a system different from modern Western medicine and current research is mainly focusing on clarifying its neurobiological mechanisms. Extensive basic and clinical research both in animal and human models has been performed to understand the physiological and biological mechanisms of acupuncture. These modulatory mechanisms have been further researched due to rapid advancement of biological techniques. The neuro-endocrine-immune regulatory mechanisms of acupuncture are now no longer a complete black box (135).

As explained before, the pathophysiology of obesity is an imbalance of a complex multi-directional interplay of the neuroendocrine and immune system, and the microbiota and gut brain axis including multiple molecular and cellular pathways. In homeostasis these interactions are well coordinated, but in pathology this cross-talk is disturbed, as indicated in **Figure 1**. Research has revealed that the neurophysiological effect of acupuncture appears to be coordinated by the CNS activation, which is essential for the regulation of the autonomic nervous system and consequently for hormonal and neuroimmune regulation (136). Based on these complex mechanisms, experimental and clinical studies suggest that acupuncture could serve as a multifaceted, multitarget regulatory therapy in the treatment of metabolic disorders, like obesity. Several reviews have found evidence of the effectiveness of acupuncture for the treatment of obesity as well as evidence for its underlying neuro-endocrine mechanisms (30–33, 48, 51, 137–139). Acupuncture involves multiple therapeutic mechanisms, including regulation of neuropeptides and neurotransmitters, inhibition of hypothalamus-pituitary-adrenal (HPA) axis hyperactivity and an immune-modulating action through a reduction in levels of proinflammatory cytokines (140, 141). Here, the literature is reviewed, summarising acupuncture’s neuro-endocrine mechanism, its effect on the CNS and on hormones such as ghrelin and leptin, its effect on the immune system and chronic low-grade inflammation, as well as on the gut microbiota and gut-brain axis. First animal studies will be discussed, followed by clinical human studies. It is important to note that there is a strong heterogeneity in the presented studies with different acupuncture techniques varying from manual acupuncture (MA), electroacupuncture (EA), auricular acupuncture and moxibustion treatment. EA is a form of acupuncture where acupoints are stimulated through a weak electric current that pass through the acupuncture needle whereas in auricular acupuncture acupoints in the ear are used (142) Moxibustion is an external heat therapy on acupoints (143). Also, the treated acupoints differed between studies and a specific explanation of what an acupoint is (144) and of the individual points or its combination is not subject of this review. Different techniques and variation between points, treatment duration and frequencies of EA and different techniques of moxibustion might exert a distinct mode of action and might have another outcome. This is further discussed in the limitations section.

## Acupuncture and experimental studies: proposed mechanisms

### Neuroendocrine effect of acupuncture and glucose and lipid metabolism

Multiple pathways have been studied to understand the underlying mechanism of acupuncture to counter obesity. Evidence showed that acupuncture regulates the secretion of related biomolecules (lipid hormones, neuromodulators, peptide hormones, and neurotransmitters), modulates inflammation, suppresses appetite, regulates lipid metabolism, and promotes browning of white adipose tissue (37, 133, 145).

#### Hypothalamus

As mentioned previously, the centre of appetite regulation is under the dominant and central control of the CNS, mainly the complex circuit in the ARC of the hypothalamus, and receives afferent and efferent signals from the brainstem and peripheral tissues, such as adipose tissue, pancreas, and stomach. Several experimental studies have revealed the regulatory effect of acupuncture on the body’s functions through modulating the neuro-endocrine network (19, 133, 146). By stimulating the neuroendocrine system, acupuncture can regulate the release of neuropeptides, neurotransmitters and hormones, and modulate relevant molecules of metabolism in obese individuals (133). In several animal studies EA has been proven effective to restrict caloric intake and reduces body weight through increasing the expression of POMC and increasing anorexigenic a-MSH peptide as well as reducing the expression of NPY and AgRP (147–150). Recently, a diet induced obese (DIO) rat model revealed that EA treatment of the acupoints ST36, CV4, CV12 and ST40 promoted the protein expression of hypothalamic SIRT1, which downregulated gene expression of NPY and upregulated that of POMC (34). Another recent animal study investigated how EA treatment of acupoints ST25, CV12, SP6 and ST36 reduces weight through several hypothalamic signaling pathways (35). These findings support the hypothesis that acupuncture suppresses caloric intake and improves IR and obesity through a central mechanism, mainly located in the ARC of the hypothalamus. However, evidence from clinical studies in humans is needed to prove this hypothesis.

#### Regulatory effect of acupuncture on appetite-regulating hormones and glucose metabolism

Acupuncture is believed to induce loss of appetite through regulating appetite-regulatory hormones like leptin, ghrelin, insulin, and cholecystokinin (CCK). An experimental study performed in high-fat-diet (HFD)-induced obese rats shows a body weight reduction and significantly decreased plasma leptin levels after EA treatment of the acupoints ST36 and ST44 (151). Acupuncture seems to reduce leptin serum levels, increases binding between leptin and its receptor, and increases leptin receptor expression in the hypothalamus as well. This suggests that the body weight loss is due to the EA-induced improvement of leptin sensitivity (36, 151). In a study with DIO rats treating acupoints ST36 and SP6, the EA-induced weight loss was due to reduced caloric intake. The underlying mechanisms may be that the orexigenic peptides NPY in the hypothalamus and ghrelin in the stomach were downregulated (147). Moreover, murine and rat models revealed that the reduction of insulin and the improvement of insulin sensitivity might be another explanatory mechanism of acupuncture-induced weight loss (152). In addition, studies show that EA induces hypoglycemic response by stimulating the cholinergic nerves and whole-body glucose uptake was increased by EA activating the parasympathetic and SNS. The most used acupoints in these studies are: ST36, ST40, LI11, CV3, CV4, CV12, ST25 (148, 153–155). Acupuncture can be effective in treating obesity, but the complex neuroendocrine interplay and the mechanism of action of acupuncture at a molecular level should be further explored. The actions of serum leptin on the hypothalamus and the correlations between CCK, insulin and other peripheral hormones as well as its exact relationship with hypothalamus is partly understood and remains to be further researched (148). It also requires further clinical validation in humans.

#### Effect of acupuncture on lipid metabolism and “browning of white adipose tissue.”

Obesity is a risk factor for the development of NAFLD and weight control is therefore crucial. A mice-model study shows that lipid absorption in the small intestine was inhibited by acupuncture, suggesting the clinical improvement of NAFLD patients (37). Another study in obese rats shows that EA treatment, of the acupoints ST36 and ST44, activated AMP-activated protein kinase (AMPK) and thereby attenuated hepatic lipid accumulation (156). Indeed, beneficial effects on cardiometabolic parameters such as plasma cholesterol and glucose have been reported upon acupuncture. Application of EA in obese rats improved the lipid profile with a reduction of lower levels of HDL and LDL as well as decreased cholesterol level (152). A possible explanation for this beneficial effect of acupuncture on the lipid metabolism is that its lipolytic effect and inhibition of lipogenesis is caused by the secretion of β-endorphin and leptin promoted by acupuncture (157).

**Figure 2:**
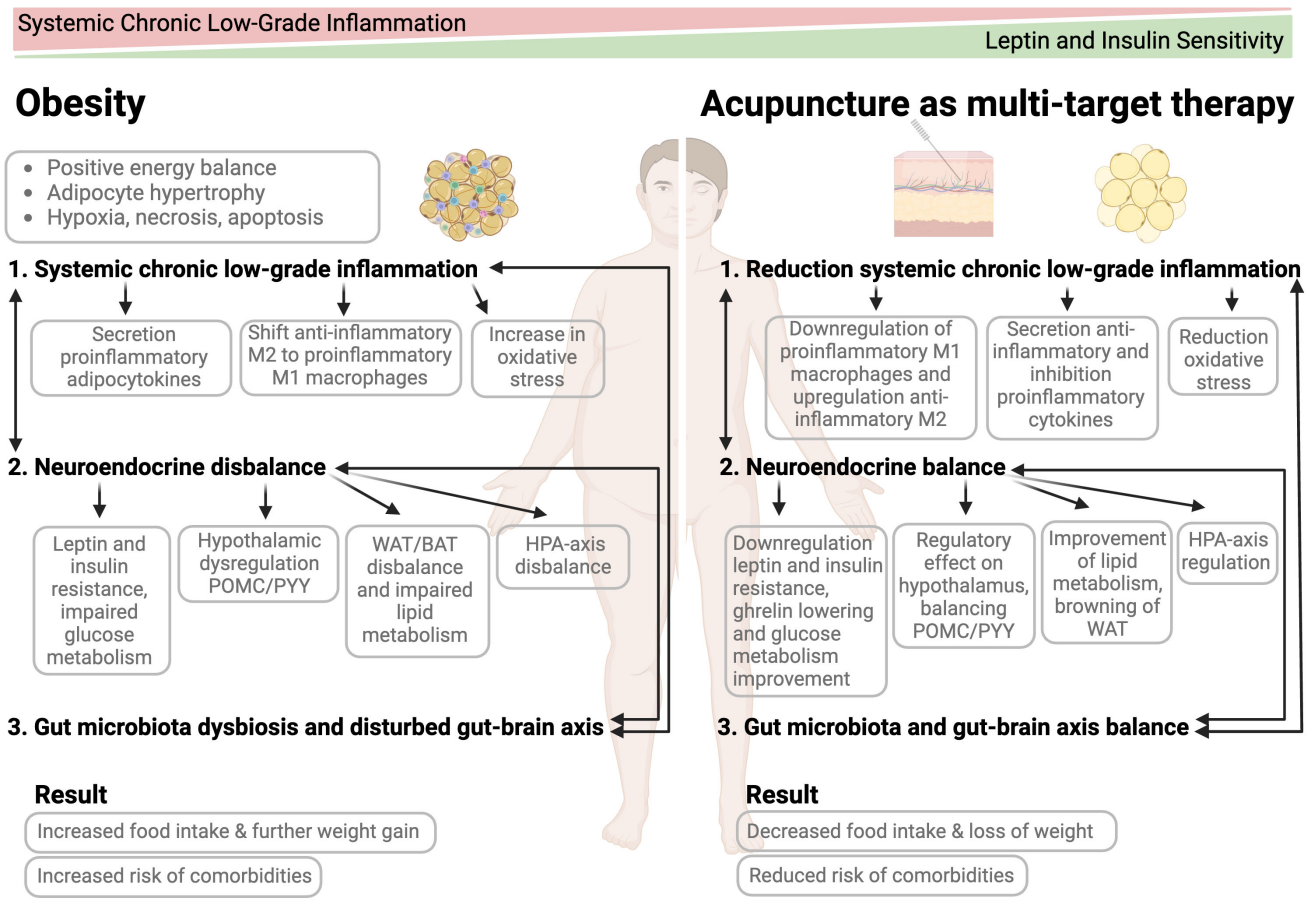
Mode of action of acupuncture as a multi-target treatment for obesity management.

“Browning” of WAT, also known as “beiging”, has become a new target for the treatment of obesity. WAT depots develop features of BAT and become energy-releasing adipocytes instead of energy storing (76, 158). Various stimuli such as the SNS, lifestyle and cold exposure can enhance non-shivering thermogenesis and contribute to the activation of BAT to burn fat and sugars to generate heat (159). But does acupuncture stimulate the browning of WAT? EA has, in fact, also been shown to be effective in WAT browning, as recent murine models have shown (38, 39, 160). EA enhances sympathetic nerve activity and remodels WAT to BAT by promoting mRNA and protein expression of uncoupling protein 1 (UCP-1). Moreover, for browning WAT, Sirtuin 1 (SIRT1), an NAD+-dependent deacetylase, plays a pivotal role. Through promoting the expression of UCP-1, SIRT1 and other fat browning proteins, adipose tissue plasticity is being induced and chronic low-grade inflammatory state modulated. The most used points in these studies are CV3, CV4, CV12, ST25, CV4, ST36, ST40, ST44 and SP6. (38–40, 145, 160). These may be possible mechanisms of action and anti-obesity targets of acupuncture as shown in **Figure 2**. Moreover, in another mice model EA, treating acupoint ST25, has shown to exert an action on two underlying anti-obesity effects: lipolysis and thermogenesis (41). It has been shown that the SNS plays a crucial role in lipolysis and the sympathetic associated macrophage (SMA) was inhibited. In addition, the regulating immunologic balance was elucidated, pointing to a potential neuro-immune cross-talk (41). The immuno-modulating effect of acupuncture is discussed below.

#### Effect of acupuncture on stress and HPA-axis

The neuroendocrine and autonomic responses linking obesity and stress are important targets for the treatment of stress-induced obesity. Acupuncture involves multiple mechanisms by which it exerts its anti-stress action. Experimental studies showed that acupuncture regulates anti-inflammatory pathways (reduction of proinflammatory cytokines), as well as neurotransmitters and neuropeptides; it regulates the autonomic nervous system, promotes signaling pathways and may inhibit the hyperactivity of the HPA-axis induced by stress stimulation (133, 140, 141). A rodent model suggests that the stress-response is regulated by acupuncture through promoting “glucocorticoid” receptor (GR) protein expression, inhibition of CRH, downregulation of ACTH level and subsequent decreased GC level. This study shows that the regulation of the HPA-axis trough EA, treating acupoints SP6, KI9, LR14 and BL23, is one of the explanatory mechanisms by which acupuncture modulates the stress response (161). A recent animal study has shown that EA at acupoint PC6 regulates the neuroendocrine and autonomic reaction induced by stress (162).

### Acupuncture action on immune system and chronic low-grade inflammation

As discussed before, the immune system and chronic low-grade inflammation play an essential role in the pathophysiology and development of obesity (163). Mounting evidence has gradually recognised that acupuncture modulates and regulates the immune system in multiple systems and multiple diseases. This non-invasive technique has emerged as a promising treatment for immunomodulation (133, 135, 145, 164–166). In murine models, general anti-inflammatory effects of acupuncture involve the regulation of multiple types and functions of the innate immune system, including macrophages, mast cells and granulocytes, as well as the adaptive immune system, such as lymphocytes. Several animal studies show that the pro-inflammatory M1 macrophages are downregulated and the anti-inflammatory M2 macrophages are upregulated through MA and EA on ST36 (167). This leads to the associated release of anti-inflammatory cytokines like TNF-β, IL-10, and the inhibition of the expression of pro-inflammatory cytokines such as TNF-α, IL-6, and IL-1, and promotes the expression of anti-inflammatory tissue repair factors. Apart from macrophages, acupuncture also regulates mast cells and the quantity of neutrophils and increases the activity of NK cells. Acupuncture controls the adaptive immune system by regulating the lymphocyte Th-cell balance as well as adaptive immune cytokines to initiate the immune response and repair damage (41, 164, 167–169). Acupuncture also seems to regulate oxidative stress, which is associated with chronic inflammation in the adipose tissue. In a recent metanalysis of 12 animal studies, acupuncture appears to reduce oxidative stress through the activation of the antioxidant enzyme system and lowering of lipid peroxidation (170). Another rat model shows that acupuncture, treatment of acupoints GB34, LR3, ST36 and SP10, exerts an antioxidant action and showed neuroprotective properties (171).

Moreover, the hypothalamus is crucial for both innate and adaptive immunity. Acupuncture has also shown to be effective to reduce neuro-inflammation through the inhibition of the activation of glial cells (astrocytes, microglia and oligodendrocytes). More specifically for obesity and obesity metabolic related disorders like T2DM, acupuncture regulates several signaling pathways affecting the production of inflammatory cytokines to inhibit inflammation. In a review of Li et al., several animal studies report the effect of acupuncture on DIO rats. The most used acupoints were ST36, ST37, ST39, SP6, LI4, LR3, CV4, KI1, ST40, GB34 (164). Among other signaling pathways, it downregulates the pro-inflammatory factor TNF-α and inhibits the macrophage proliferation and infiltration of macrophages into adipose tissue and, in turn, stimulates the anti-inflammatory adipokine balance lowering the adiponectin/leptin ratio. In addition, the chronic low-grade inflammation is reduced, and insulin sensitivity and glucose tolerance improved, and blood lipid content lowered (164).

The CNS plays a pivotal role in integration of acupuncture-driven information. Several main regulatory pathways can be distinguished through which acupuncture exerts its anti-inflammatory effect: cholinergic anti-inflammatory pathway (CAIP), vagal-adrenal medulla-dopamine pathway, spinal sympathetic pathway, HPA-axis, and the brain-gut axis (BGA). First, looking at the cholinergic anti-inflammatory pathway (CAIP), upon adrenalectomy and vagotomy, the anti-inflammatory effect of EA was blocked, implying the anti-inflammatory effect of the activation of the CAIP. Second, in relation to the vagal-adrenal medulla-dopamine pathway, EA can modulate body-physiology through somatosensory autonomic reflexes. These reflexes start with the stimulation of peripheral nerves followed by transmission of sensory information to the brain and subsequent activation of autonomic pathways such as inhibiting systemic inflammation (164, 172). A High-Fat-Diet (HFD) induced rat model examined the relation between the autonomic nervous system and the anti-inflammatory effect of EA treatment of acupoint ST36. Here it was concluded that EA inhibits low-grade inflammation and this mechanism was closely related to the cholinergic anti-inflammatory pathway as well as enhancement of the vagal activity (42). It has been demonstrated that through acupuncture signals the vagal-adrenal medulla reflex is being triggered and dopamine is being released, exerting an anti-inflammatory effect. Neurons innervating the deep hindlimb fascia, the so-called PROKR2^Cre^-marked sensory neurons, are fundamental for driving the vagal-adrenal axis (173). Experimental studies show that low-frequency EA of acupoint ST36 stimulates dopamine secretion, modulating vagal activity and thereby exerting an anti-inflammatory effect (174). Catecholamines from the adrenal gland are released by acupuncture stimulation and by acting on peripheral dopamine D1 receptors produce systemic anti-inflammatory effects (175). Moreover, the spinal sympathetic pathway plays an important role because its activation through acupuncture has an anti-inflammatory action. After sympathectomy the anti-inflammatory effect of acupuncture significantly reduces, also indicating a role of the latter pathway. Furthermore, it has been shown that acupuncture bi-directionally regulates the HPA-axis restoring the normal anti-inflammatory action. One of the possible explanations is that acupuncture exerts its anti-inflammatory effect through the regulation of intestinal flora, which is further explored below (135, 164, 168). Lastly, acupuncture exerts an effect on the bi-directional brain-gut axis (BGA). A somatosensory-autonomic reflex pathway controls intestinal inflammation, thereby restoring BGA balance. In addition, the changes in the gut microbiota are transmitted to the brain *via* the sympathetic nerves or the vagus nerve (135). This is also further reviewed below. In conclusion, accumulated evidence shows that acupuncture exerts a strong anti-inflammatory effect and reduces chronic low inflammation as shown in **Figure 2**, and human studies are needed to validate these findings.

### Effect of acupuncture on gut microbiota and gut-brain axis

#### Acupuncture and the gut microbiota

Recent research in both animal models and humans has focused on the possible role of the gut microbiota in acupuncture, as acupuncture can potentially regulate the neuroendocrine-immune system of the body and improve the intestinal microenvironment. Several animal studies suggest that acupuncture may affect the gut microbiota, and this may play a role in the effectiveness of acupuncture (43–45, 176–179). In obese male rats, EA decreased the Firmicutes-to-Bacteroidetes ratio compared to obese rats who did not undergo acupuncture (43). The microbiota profile at genus level after eight weeks of acupuncture treatment was similar to that of a control group on a normal diet (43). Mice on a high-fat diet (HFD) treated with EA showed increased gut bacterial diversity over time (46). Similarly, mice on a HFD treated with daily EA for 21 and 28 days showed higher alpha-diversity after treatment compared to the gut microbiota of obese control mice (45). In addition to improved body weight, serum leptin, lipid, and adiponectin levels after EA, a potential action mechanism is described of acupuncture *via* the ENS (45). In a more recent study, EA for 21 days in high-fat DIO mice rescued dysbiosis of the gut microbiota in the cecum (179). Moreover, acupuncture treatment has been linked to inhibition of gut dysbiosis in various animal models, including in male mice models for IBS (180) chronic colitis (181), T2DM (44), Parkinson’s disease (176) and insomnia (177). In male rat models with post-inflammatory irritable bowel syndrome, acupuncture and moxibustion treatment were independently associated with changed gut microbiota (178, 182). Combined, this evidence potentially underscores an association between acupuncture and changes in gut microbiota in several disease models, including but not limited to obesity and disorders of the gastrointestinal tract, although the causality remains to be further evaluated. Specifically, the exact mechanism of action of acupuncture on the microbiota structure remains to be further evaluated but is suggested to be linked to the gut brain axis, as described below.

### Acupuncture and the gut-brain axis

Although limited, some animal studies have suggested a role for acupuncture *via* regulation of the ENS as part of the gut-brain axis. The SNS, influencing the ENS, exerts a mainly inhibitory role over the gastro-intestinal tract (115) and it is likely that its activation *via* release of stress-hormones such as epinephrine, norepinephrine and cortisol, contributes to a dysregulation of gut motility (116). Among the peripheral gastrointestinal hormones, 5-HT is an important neurotransmitter in the gut brain axis (117). Although the function of 5-HT has not yet been fully explained, several studies describe a role on secretory and peristaltic reflexes, by stimulating enteric neurons (110, 117). A study in mice suggests improved colonic motility after treatment with EA *via* regulation of the ENS, through alteration of both excitatory and inhibitory enteric neurons (183). In two rat models with diarrhea-prominent irritable bowel syndrome (IBS-D), EA at ST25, ST36 and LR3 alleviated IBS-D symptoms and lowered the levels of NPY and 5-HT in the gut brain axis, suggesting restored balance of the gut brain axis (184). Moreover, in adult patients with constipation dominant-IBS (IBS-C), an RCT comparing EA to mild-warm moxibustion treatment at ST25 and ST37 showed improvement of defecation frequency compared to baseline up to three months after treatment, and the effects of EA were proposed to be mediated through alleviation of visceral hypersensitivity and improved brain-gut function as visualised with fMRI (185). Additionally, several studies in animals and humans show altered levels of stress hormones such as cortisol and epinephrine, and changes in 5-HT associated with acupuncture, although mostly in the context of visceral pain management (186–189). In mice and rats with IBS-associated visceral hypersensitivity, treatment with EA significantly reduced 5-HT and 5-HT receptor mRNA expression levels compared to sham-treatment and was associated with attenuated visceral hypersensitivity (186, 187). In the context of obesity, a potential role for 5-HT on thermogenesis of brown adipose tissue in mice has been reported (189) and gut microbiota are directly implicated in the process of peripheral 5-HT biosynthesis in their host, both animals and human adults with obese phenotypes (122, 190, 191). However, the clinical relevance and changes in these hormonal levels related to acupuncture treatment have not (yet) been studied in the context of acupuncture treatment in mice or humans with obesity.

## Acupuncture clinical human studies

### Neuroendocrine system

In human trials acupuncture emerges as a potentially effective complementary treatment of obesity as it is thought to induce loss of appetite, down-regulates insulin and leptin resistance, and lowers ghrelin as well as glucose levels (31, 32, 192). A systematic review of acupuncture in adults with obesity, which reviewed 11 sham-controlled RCTs published in English, suggests that acupuncture, including EA, ear acupuncture and MA, was indeed effective in reducing BMI, hip- and waist circumference, body fat mass, but not body weight. The most used acupoints are LI4, LI11, ST25, ST36, ST44, GB28, CV4, CV9, CV12, SP6, LV3 (193). Due to the limited sample size, heterogeneity and varying methodologic quality, further assessments are necessary. Acupuncture treatment of acupoints LI4, ST36, ST44, SP6, ST25, GB28, CV4, CV6, CV12, SP6, LI11, ST40, SP9 alone or combined with low-calorie diet had beneficial effects on serum leptin levels (52, 194). Due to methodological limitations, lack of homogeneity of the included studies and small effect sizes, these findings should be taken with caution. Park et al. included eight RCTs in their systematic review and concluded that a combination of acupuncture and diet therapy or exercise was more effective than diet and exercise alone in reducing serum leptin levels. Between verum acupuncture and sham acupuncture, however, there was no significant difference, although compared to oral anorexiant therapy or no treatment, acupuncture was proven to be more effective. Again, due to methodological limitations, the results should be used with care (32). In a systematic review and meta-analysis of 20 RCTs Wu et al. showed that acupuncture could be a viable option in treating IR by reducing fasting insulin (FINS) levels, fasting blood glucose (FBG), and 2h postprandial blood glucose (2hPG). Methodological flaws, however, limit these conclusions (47, 71). A small clinical study of 16 obese women showed that EA treatment of acupoints BL18, BL20, BL21, BL23, LI10, LI11, ST36 was effective in reducing glucose level, whereas auricular acupuncture did not (195). This finding is in line with the animal models where EA was shown to act on the glucose metabolism. Again, this study has limitations as it was an uncontrolled pilot study. On the other hand, a systematic review of 33 RCTs performed by Chen et al. concludes that the effects of acupuncture on blood glucose are uncertain. They did report a significant, albeit small reduction of body weight, BMI and waist circumference compared to the non-acupuncture groups. Furthermore, reduction of serum lipid parameters has been found (48). The evidence is of moderate to very low quality and well-designed large trials with long-term follow up are needed.

### Chronic low-grade inflammation

Although there is substantial animal evidence, human trials studying the effects of acupuncture and chronic low-grade inflammation in obesity are limited. Acupuncture has shown to be effective in several inflammatory disorders, like inflammatory bowel disease (IBD), effectively regulating the inflammatory factors (196). An RCT reviewing EA performed in obese women noted no difference in the high-sensitivity C-reactive protein (hs-CRP) levels, but positive effects in the anti-heat shock protein (anti-Hsp), concluding that acupuncture exerts immunomodulatory effects on the human immune system but not anti-inflammatory effects. In a sham-controlled RCT of 196 obese subjects a significant decrease in preoxidant-antioxidant balance (PAB) was found in the treatment group (acupuncture combined with low-calorie diet) compared to the control group (diet alone). Here the EA treatment used acupoints ST25, GB28, CV6, CV9, CV12, SP6, LI11, ST40, SP6, SP9. This is in line with animal studies showing that oxidative stress is reduced by acupuncture (197).

### Gut Microbiota and gut brain axis

As described previously, some evidence in animal models of acupuncture on obesity and modulation of gut microbiota composition is presented. In humans, the evidence is still scarce but emerging changes in species abundance and diversity of the gut microbiome have been documented in patients undergoing treatment with EA (49, 53, 198). In perimenopausal women with abdominal obesity, EA for eight weeks in combination with diet decreased the abundance of the species and increased the species diversity of gut microbiota (49). After EA treatment, notably the two species Klebsiella and Kosakonia were increased in perimenopausal patients (49). In adult studies using acupuncture for various conditions, emerging evidence points towards an effect of acupuncture on the gut microbiome *via* the gut brain axis. A recent sham-controlled study in adults with antipsychotic-related constipation treated with EA showed that richness of various bacterial strains such as Klebsiella and Gammaproteobacteria was significantly higher after acupuncture, and was associated with significantly increased spontaneous bowel movements and reduced use of rescue medication. Moreover, Firmicutes-to-Bacteroidetes ratio was increased post-treatment (198). In a population of patients with knee osteoarthritis, eight weeks of EA modified gut microbiota diversity and microbiota richness of species (53). In healthy adults, a recent study on acupoint application treatment - a non-invasive modality of traditional acupuncture - showed increased Firmicutes/Bacteroidetes ratio after three sessions in 24 months compared to sham control group (199). It should be noted that true clinical consequences of the changes in gut microbiota in obese patients are to date not established in humans. As described previously in the section “Microbiota dysbiosis and gut-brain axis”, changes in diversity and richness of the microbiome have been linked to many significant biological changes with consequences for human health (106, 107); in the context of obesity, a dysbiotic Bacteroidetes/Firmicutes ratio is frequently reported (107, 125). However, the clinical relevance of the currently presented changes in microbiota profiles after treatment remain to be evaluated. Indeed, this “signature” dysbiosis is mainly shown in rodent models, as studies in humans report varying results regarding microbiota profiles (128, 129, 200), which could be the result of methodological differences between studies and differences in gut microbiota sample processing (99). The varying duration of interventions, use of different acupoints, and the variety of sex and age distribution potentially impact the results. In acupuncture studies, including microbiota profiles in animal models, mainly male rodent models are used. In the few studies in adults, sex distribution differs between studies, ranging from women only (49) to two-thirds of the population subgroups being women (53, 198, 199). Recent studies reported that changes in the gut microbiota associated to metabolic illnesses are different in men and women (201) and this could be translated to acupuncture treatment (50). To the best of our knowledge, no studies analysing acupuncture in combination with gut microbiota analysis in children have been performed to date. This evidence combined, although limited, suggests that acupuncture may affect constituents of the brain-gut axis, including the enteric and autonomic nervous system and the gut microbiota, and this potential mechanism of action should be investigated further when evaluating acupuncture in patients with metabolic diseases.

### Acupuncture combined with lifestyle intervention.

An adequate strategy to reduce weight is a Very Low-Calorie Diet (VLCD) (202). As mentioned before, however, losing weight is not as easy as it seems as adherence to diet is low and calorie restriction might increase cortisol levels and perceived stress (203). As previously discussed, acupuncture seems to reduce appetite through its neuro-endocrine regulation and reduces cortisol levels and stress. However, acupuncture alone seems not to be clinically effective on body weight loss (33). Therefore, a combined intervention of VLCD and acupuncture is proposed. In line with previous research (31, 32, 48, 192) acupuncture combined with lifestyle modification (diet alone or diet in combination with physical exercise) showed greater reduction of body weight than acupuncture alone. Also, greater serum leptin levels reduction was found when combining acupuncture with diet (32). A synergetic effect between lifestyle modifications and acupuncture is therefore suggested and this combined intervention may be a more effective and viable option. Recently, a large-scale retrospective chart review was performed of 11,233 obese patients with combined acupuncture and VLCD underscored that the combination can induce weight reduction in obese patients (50). However, due to the lack of a control group, a prospective trial is needed to validate these findings and therefore such RCT is currently executed.

### Safety and cost-effectiveness of acupuncture

Outcomes of systematic reviews on the effectiveness and safety of acupuncture both in adults and in children support the notion that acupuncture is well-tolerated and can be considered a safe intervention (48, 139, 192, 204, 205, 131). The frequency of reported adverse events (AEs) associated with adult and pediatric needle acupuncture is low and most AEs are mild and transient. Reported AEs include mild ecchymosis, bleeding, pain upon needle insertion,dizziness caused by acupuncture and EA and mild skin burns caused by moxibustion (48, 139, 204, 205). Another noteworthy factor when considering acupuncture for obesity is the potential cost-effectiveness. Worldwide, obesity represents a significant economic burden in developed as well as developing countries (206). Although a cost-effectiveness analysis has not yet been performed in the context of obesity, several cost-effectiveness analyses of acupuncture versus usual care for varying conditions (dysmenorrhea, allergic rhinits, osteoarthritis, headache and acute lower back pain) show a benefit for this treatment modality (207, 208). Its cost-effectiveness should be further evaluated in the specific case of obesity, especially considering the current treatment options ranging from dietary changes and prescription medication to bariatric surgery.

### Limitations and future direction

In conclusion, acupuncture might be an effective, safe and cost-effective method for obesity. However, the results remain controversial and conclusions should be taken with caution. There are several limitations in the studies regarding presented obese animal models. First, the treated acupoints differed between studies and an explanation for the use of these points was not at all or only partially provided in most studies, basing it on a proposed transposition model of acupoints in rodents and referring to the points used in a human trial by who based the points on expert opinion. Different acupoints might regulate different biological pathways and a combination of points may activate several pathways simultaneously and standardization is lacking. Furthermore, frequencies of EA, durations of the treatment and wave types varied as well as different moxibustion techniques were applied. Among others, the most used acupoints in both animal and human studies in the context of obesity are ST25, ST36, CV9, CV12, CV4, SP6, ST44 (146). The exact mechanism of the single actions of the points and the combination of points, however, must be further elucidated. The myriad of studies thus has individual variabilities which makes it difficult to evaluate systematic reviews or meta-analyses for their accuracy and applicability to clinical practice. With regards to human studies, the quality of most included RCTs is low due to high heterogeneity of interventions (MA, EA, auricular acupuncture, moxibustion) different points were used and studies used with and without diet and/or exercise. Also, inclusion criteria (gender, age, BMI) and studied endpoints differed widely. Most studies that have been reviewed are low in sample size, have a short follow-up and overall high risk of bias. Moreover, there is a lack of consensus of general accepted criteria and international accepted protocols regarding the acupoint selection and technique, caused by a strong doctor variability, and existence of numerous acupuncture traditions. There is also a publication-geographical bias as many RCTs were conducted in China and the question remains whether these findings may be extrapolated to the rest of the world. Due to these methodological flaws the real effects may be distorted and quality of the evidence is weak preventing us from drawing definitive conclusions. Lastly, there is a continuous academic discussion about the use of sham-acupuncture for placebo-controlled studies. Acupuncture is often criticised for having a placebo effect. Compared to “real acupuncture”, sham techniques might exert similar biological effects and its use should be reconsidered (209). These limitations make acupuncture an avenue for treatment but do yet not support mainstream treatment until further rigorous research is conducted in this field. Future research should try to overcome these methodological limitations and therefore well-designed rigorous RCTs and multi-centre trials with large sample size and protocolized standard treatments are needed to draw evidence-based conclusions about the beneficial effect of acupuncture as a treatment for obesity. Furthermore, the understanding of acupuncture’s action mechanism may be further researched thanks to the advancements in systems biology, modern omics technology and emerging techniques to analyse biological information such as tracer technology, i-needles, metabolomics, two-photon technology, and cryo-electron microscopy technology. This will enable acupuncture’s action on the neuro-endocrine-immune network to be elucidated (133, 175). This progress in biomedical research combined with future clinical trial should shed greater light on acupuncture as a viable treatment option for obesity. Lastly, research should also be focused on the accessibility and equity of acupuncture practice. For multiple reasons acupuncture implementation has been limited due to fact of the limitations in the insurance reimbursement as well as the fear for needles as a potential barrier (210).

## Conclusion

Obesity is a multifactorial and multiorgan disease, and its pathophysiology can be explained by a complex orchestra of a neuro-endocrine crosstalk, chronic low-grade inflammation, and microbiota dysbiosis. The patho-physiology creates one vicious cycle leading to another, with obesity leading to chronic low-grade inflammation, enhancing gutmicrobiota dysbiosis, insulin and leptin resistance. In this review the interplay of these seemingly independent systems have been reviewed. Despite its apparent simplicity, the solution to losing weight is not as easy as it seems, and the search for other options other than lifestyle modification, medication and bariatric surgery is ongoing. Modern medical research mainly relies on a reductionist approach, studying single systems and components of the problem on a molecular level. A viable “new” treatment option is acupuncture and has been the subject of our narrative review. So far, the mechanisms for the beneficial effects of acupuncture for obesity management have been revealed by several experimental animal studies including regulation of the neuro-endocrine system, low-grade inflammation and gut microbiota brain axis. In light of the presented findings, acupuncture might be an effective, safe, and cost-effective therapeutic strategy for obesity. These results on the functional mechanism, however, have not yet been fully extrapolated and validated in human studies, and due to the numerous limitations in the conducted research, available findings in humans are inconclusive and results scarce. Thus, there is a strong urge for more careful analysis and further rigorous prospective comparative research. A combined treatment with acupuncture and diet shows promising results and will be the subject of future research by our group.

## Author contributions

All authors critically reviewed the paper, RGL bears full responsibility for the content. All authors contributed to the article and approved the submitted version.

## References

[1] Boutari C Mantzoros CS. A 2022 update on the epidemiology of obesity and a call to action: as its twin COVID-19 pandemic appears to be receding, the obesity and dysmetabolism pandemic continues to rage on. Metabolism (2022) 133. doi: 10.1016/j.metabol.2022.155217 PMC9107388 35584732

[2] Valenzuela PL Carrera-Bastos P Castillo-García A Lieberman DE Santos-Lozano A Lucia A. Obesity and the risk of cardiometabolic diseases. Nat Rev Cardiol (2023) 20(7):475–94. doi: 10.1038/s41569-023-00847-5 36927772

[3] Jaacks LM Vandevijvere S Pan A McGowan CJ Wallace C Imamura F. The obesity transition: stages of the global epidemic. Lancet Diabetes Endocrinol (2019) 7:231–40. doi: 10.1016/S2213-8587(19)30026-9 PMC7360432 30704950

[4] World Health Organization. WHO. (2023). Available at: https://www.who.int/news-room/fact-sheets/detail/obesity-and-overweight (Accessed 19 April 2023).

[5] World Health Organization. Regional Office for Europe. (2022). WHO European Regional Obesity Report 2022. World Health Organization. Regional Office for Europe. Available at: https://apps.who.int/iris/handle/10665/353747.

[6] NCD Risk Factor Collaboration (NCD-RisC). Trends in adult body-mass index in 200 countries from 1975 to 2014: a pooled analysis of 1698 population-based measurement studies with 19·2 million participants. Lancet (2016) 387:1377–96. doi: 10.1016/S0140-6736(16)30054-X PMC7615134 27115820

[7] Lobstein T Jackson-Leach R Powis J Brinsden H Gray M. World obesity atlas 2023 (2023). Available at: www.johnclarksondesign.co.uk.

[8] Chooi YC Ding C Magkos F. The epidemiology of obesity. Metabolism (2019) 92:6–10. doi: 10.1016/j.metabol.2018.09.005 30253139

[9] Guembe MJ Fernandez-Lazaro CI Sayon-Orea C Toledo E Moreno-Iribas C Cosials JB. Risk for cardiovascular disease associated with metabolic syndrome and its components: a 13-year prospective study in the RIVANA cohort. Cardiovasc Diabetol (2020) 19:195. doi: 10.1186/s12933-020-01166-6 33222691 PMC7680587

[10] Rinella ME Lazarus JV Ratziu V Francque SM Sanyal AJ Kanwal F. A multi-society Delphi consensus statement on new fatty liver disease nomenclature. J Hepatol (2023). doi: 10.1016/j.jhep.2023.06.003 37983810

[11] González-Muniesa P Mártinez-González MA Hu FB Després JP Matsuzawa Y Loos RJF. Obesity. Nat Rev Dis Primers (2017) 3:17034. doi: 10.1038/nrdp.2017.34 28617414

[12] Speakman JR. Evolutionary perspectives on the obesity epidemic: adaptive, maladaptive, and neutral viewpoints. Annu Rev Nutr (2013) 33:289–317. doi: 10.1146/annurev-nutr-071811-150711 23862645

[13] Heymsfield SB Wadden TA. Mechanisms, pathophysiology, and management of obesity. New Engl J Med (2017) 376:254–66. doi: 10.1056/NEJMra1514009 28099824

[14] Dein S. The myth of the golden past: Critical perspectives on the paleo diet. Anthropology Food (2022). doi: 10.4000/aof.13805

[15] van der Valk ES van den Akker ELT Savas M Kleinendorst L Visser JA Van Haelst MM. A comprehensive diagnostic approach to detect underlying causes of obesity in adults. Obes Rev (2019) 20:795–804. doi: 10.1111/obr.12836 30821060 PMC6850662

[16] Barber TM Kyrou I Randeva HS Weickert MO. Mechanisms of insulin resistance at the crossroad of obesity with associated metabolic abnormalities and cognitive dysfunction. Int J Mol Sci (2021) 22:546. doi: 10.3390/ijms22020546 33430419 PMC7827338

[17] Lin X Li H. Obesity: epidemiology, pathophysiology, and therapeutics. Front Endocrinol (Lausanne) (2021) 12:706978. doi: 10.3389/fendo.2021.706978 34552557 PMC8450866

[18] Martins C Nymo S Coutinho SR Rehfeld JF Hunter GR Gower BA. Association between fat-free mass loss, changes in appetite, and weight regain in individuals with obesity. J Nutr (2023) 183(5):1330–37. doi: 10.1016/j.tjnut.2023.03.026 36963504

[19] Zhang K Zhou S Wang C Xu H Zhang L. Acupuncture on obesity: clinical evidence and possible neuroendocrine mechanisms. Evid Based Complement Alternat Med (2018) 2018:6409389. doi: 10.1155/2018/6409389 30013603 PMC6022277

[20] Bray GA Frühbeck G Ryan DH Wilding JPH. Management of obesity. Lancet (2016) 387:1947–56. doi: 10.1016/S0140-6736(16)00271-3 26868660

[21] Wilding JPH Batterham RL Calanna S Davies M Van Gaal LF Lingvay I. Once-weekly semaglutide in adults with overweight or obesity. N Engl J Med (2021) 384:989–1002. doi: 10.1056/NEJMoa2032183 33567185

[22] Rosen CJ Ingelfinger JR. Shifting tides offer new hope for obesity. N Engl J Med (2022) 387:271–3. doi: 10.1056/NEJMe2206939 35657317

[23] Jastreboff AM Aronne LJ Ahmad NN Wharton S Connery L Alves B. Tirzepatide once weekly for the treatment of obesity. N Engl J Med (2022) 387:205–16. doi: 10.1056/NEJMoa2206038 35658024

[24] Lingvay I Brown-Frandsen K Colhoun HM Deanfield J Emerson SS Esbjerg S. Semaglutide for cardiovascular event reduction in people with overweight or obesity: SELECT study baseline characteristics. Obesity (2023) 31:111–22. doi: 10.1002/oby.23621 PMC10107832 36502289

[25] Li M-F. Rise and fall of anti-obesity drugs. World J Diabetes (2011) 2:19. doi: 10.4239/wjd.v2.i2.19 21537456 PMC3083904

[26] James WPT Caterson ID Coutinho W Finer N Van Gaal LF Maggioni AP. Effect of sibutramine on cardiovascular outcomes in overweight and obese subjects. N Engl J Med (2010) 363:905–17. doi: 10.1056/NEJMoa1003114 20818901

[27] Christensen R Kristensen PK Bartels EM Bliddal H Astrup A. Efficacy and safety of the weight-loss drug rimonabant: a meta-analysis of randomised trials. Lancet (2007) 370:1706–13. doi: 10.1016/S0140-6736(07)61721-8 18022033

[28] Przegaliński E Witek K Wydra K Kotlińska JH Filip M. 5-HT2C receptor stimulation in obesity treatment: orthosteric agonists vs. allosteric modulators. Nutrients (2023) 15:1449. doi: 10.3390/nu15061449 36986191 PMC10058696

[29] Chang S-H Stoll CRT Song J Varela JE Eagon CJ Colditz GA. The effectiveness and risks of bariatric surgery: an updated systematic review and meta-analysis, 2003-2012. JAMA Surg (2014) 149:275–87. doi: 10.1001/jamasurg.2013.3654 PMC3962512 24352617

[30] Cho S-H Lee J-S Thabane L Lee J. Acupuncture for obesity: a systematic review and meta-analysis. Int J Obes (Lond) (2009) 33:183–96. doi: 10.1038/ijo.2008.269 19139756

[31] Belivani M Dimitroula C Katsiki N Apostolopoulou M Cummings M Hatzitolios AI. Acupuncture in the treatment of obesity: A narrative review of the literature. Acupuncture Med (2013) 31:88–97. doi: 10.1136/acupmed-2012-010247 23153472

[32] Park KS Park KI Suh HS Hwang DS Jang JB Lee JM. The efficacy and safety of acupuncture on serum leptin levels in obese patients: A systematic review and meta-analysis. Eur J Integr Med (2017) 11:45–52. doi: 10.1016/j.eujim.2017.03.004

[33] Kim SY Shin IS Park YJ. Effect of acupuncture and intervention types on weight loss: a systematic review and meta-analysis. Obes Rev (2018) 19:1585–96. doi: 10.1111/OBR.12747 30180304

[34] Shu Q Chen L Wu S Li J Liu J Xiao L. Acupuncture targeting SIRT1 in the hypothalamic arcuate nucleus can improve obesity in high-fat-diet-induced rats with insulin resistance *via* an anorectic effect. Obes Facts (2020) 13:40–57. doi: 10.1159/000503752 31935731 PMC7105640

[35] Leng J Xiong F Yao J Dai X Luo Y Hu M. Electroacupuncture Reduces Weight in Diet-Induced Obese Rats *via* Hypothalamic Tsc1 Promoter Demethylation and Inhibition of the Activity of mTORC1 Signaling Pathway. Evid Based Complement Alternat Med (2018) 2018:3039783. doi: 10.1155/2018/3039783 29853949 PMC5944273

[36] Li X Wu Z Chen Y Cai R Wang Z. Effect of acupuncture on simple obesity and serum levels of prostaglandin E and leptin in sprague-dawley rats. Comput Math Methods Med (2021) 2021:6730274. doi: 10.1155/2021/6730274 34646336 PMC8505091

[37] Han J Guo X Meng X-J Zhang J Yamaguchi R Motoo Y. Acupuncture improved lipid metabolism by regulating intestinal absorption in mice. World J Gastroenterol (2020) 26:5118–29. doi: 10.3748/wjg.v26.i34.5118 PMC7495030 32982113

[38] Lu S-F Tang Y-X Zhang T Fu S-P Hong H Cheng Y. Electroacupuncture reduces body weight by regulating fat browning-related proteins of adipose tissue in HFD-induced obese mice. Front Psychiatry (2019) 10:353. doi: 10.3389/fpsyt.2019.00353 31244685 PMC6580183

[39] Tang Q Lu M Xu B Wang Y Lu S Yu Z. Electroacupuncture regulates inguinal white adipose tissue browning by promoting sirtuin-1-dependent PPARγ Deacetylation and mitochondrial biogenesis. Front Endocrinol (Lausanne) (2020) 11:607113. doi: 10.3389/fendo.2020.607113 33551999 PMC7859442

[40] Luo D Liu L Liang F-X Yu Z-M Chen R. Electroacupuncture: A feasible sirt1 promoter which modulates metainflammation in diet-induced obesity rats. Evid Based Complement Alternat Med (2018) 2018:5302049. doi: 10.1155/2018/5302049 30425749 PMC6217753

[41] Lu M He Y Gong M Li Q Tang Q Wang X. Role of neuro-immune cross-talk in the anti-obesity effect of electro-acupuncture. Front Neurosci (2020) 14:151. doi: 10.3389/fnins.2020.00151 32180699 PMC7059539

[42] Jie X Li X Song J-Q Wang D Wang J-H. Anti-inflammatory and autonomic effects of electroacupuncture in a rat model of diet-induced obesity. Acupuncture medicine : J Br Med Acupuncture Soc (2018) 36:103–9. doi: 10.1136/acupmed-2016-011223 29487062

[43] Wang H Wang Q Liang C Su M Wang X Li H. HWQ, evid based complement alternat M. Acupuncture regulating gut microbiota in abdominal obese rats induced by high-fat diet. Evid Based Complement Alternat Med (2019) 2019:4958294. doi: 10.1155/2019/4958294 31275411 PMC6582896

[44] Wang H Chen X Chen C Pan T Li M Yao L. Electroacupuncture at lower he-sea and front-mu acupoints ameliorates insulin resistance in type 2 diabetes mellitus by regulating the intestinal flora and gut barrier. Diabetes Metab Syndrome Obes (2022) 15:2265–76. doi: 10.2147/DMSO.S374843 PMC9348137 35936053

[45] Dou D Chen QQ Zhong Z Xia X Ding W-J. Regulating the enteric nervous system against obesity in mice by electroacupuncture. Neuroimmunomodulation (2020) 27:48–57. doi: 10.1159/000506483 32516787

[46] Si Y-C Miao W-N He J-Y Chen L Wang Y-L Ding W-J. Regulating gut flora dysbiosis in obese mice by electroacupuncture. Am J Chin Med (Gard City N Y) (2018) 46:1481–97. doi: 10.1142/s0192415x18500763 30284469

[47] Wu L Chen X Liu Y Lan J Wu C Li Z. Role of acupuncture in the treatment of insulin resistance: A systematic review and meta-analysis. Complement Ther Clin Pract (2019) 37:11–22. doi: 10.1016/j.ctcp.2019.08.002 31445362

[48] Chen J Chen D Ren Q Zhu W Xu S Lu L. Acupuncture and related techniques for obesity and cardiovascular risk factors: a systematic review and meta-regression analysis. Acupuncture Med (2020) 38:227–34. doi: 10.1136/acupmed-2018-011646 32310001

[49] Sheng J Yang G Jin X Si C Huang Y Luo Z. Electroacupuncture combined with diet treatment has a therapeutic effect on perimenopausal patients with abdominal obesity by improving the community structure of intestinal flora. Front Physiol (2021) 12:708588. doi: 10.3389/fphys.2021.708588 34899365 PMC8656264

[50] Fumagalli M Landgraaf RG Schiavi-Lods NN Golcea SS Büller HR Nieuwdorp M. Novel insights in weight loss: acupuncture combined with low calory diet – A swiss experience. Acupunct Med (2023). doi: 10.1177/09645284231202811 PMC10656784 37789716

[51] Zhong Y-M Luo X-C Chen Y Lai D-L Lu W-T Shang Y-N. Acupuncture versus sham acupuncture for simple obesity: a systematic review and meta-analysis. Postgrad Med J (2020) 96:221–7. doi: 10.1136/postgradmedj-2019-137221 PMC7146934 32015189

[52] Darbandi S Darbandi M Mokarram P Owji A-A Zhao B Ghayor-Mobarhan M. Effects of body electroacupuncture on plasma leptin concentrations in obese and overweight people in Iran: a randomized controlled trial. Altern Ther Health Med (2013) 19:24–31. 23594450

[53] Wang T-Q Li L-R Tan C-X Yang J-W Shi G-X Wang L-Q. Effect of electroacupuncture on gut microbiota in participants with knee osteoarthritis. Front Cell Infect Microbiol (2021) 11:597431. doi: 10.3389/fcimb.2021.597431 34671567 PMC8521167

[54] Lustig RH Collier D Kassotis C Roepke TA Ji Kim M Blanc E. Obesity I: Overview and molecular and biochemical mechanisms. Biochem Pharmacol (2022) 199:115012. doi: 10.1016/J.BCP.2022.115012 35393120 PMC9050949

[55] Sáinz N Barrenetxe J Moreno-Aliaga MJ Martínez JA. Leptin resistance and diet-induced obesity: central and peripheral actions of leptin. Metabolism (2015) 64:35–46. doi: 10.1016/j.metabol.2014.10.015 25497342

[56] López M Nogueiras R Tena-Sempere M Diéguez C. Hypothalamic AMPK: a canonical regulator of whole-body energy balance. Nat Rev Endocrinol (2016) 12:421–32. doi: 10.1038/nrendo.2016.67 27199291

[57] Schwartz MW Woods SC Porte D Seeley RJ Baskin DG. Central nervous system control of food intake. Nature (2000) 404:661–71. doi: 10.1038/35007534 10766253

[58] Jais A Brüning JC. Arcuate nucleus-dependent regulation of metabolism-pathways to obesity and diabetes mellitus. Endocr Rev (2022) 43:314–28. doi: 10.1210/endrev/bnab025 PMC8905335 34490882

[59] Matafome P Seiça R. The role of brain in energy balance. Adv Neurobiol (2017) 19:33–48. doi: 10.1007/978-3-319-63260-5_2 28933060

[60] Liang F Kume S Koya D. SIRT1 and insulin resistance. Nat Rev Endocrinol (2009) 5:367–73. doi: 10.1038/nrendo.2009.101 19455179

[61] Wilson JL Enriori PJ. A talk between fat tissue, gut, pancreas and brain to control body weight. Mol Cell Endocrinol (2015) 418 Pt 2:108–19. doi: 10.1016/j.mce.2015.08.022 26316427

[62] Crujeiras AB Carreira MC Cabia B Andrade S Amil M Casanueva FF. Leptin resistance in obesity: An epigenetic landscape. Life Sci (2015) 140:57–63. doi: 10.1016/j.lfs.2015.05.003 25998029

[63] Maffei M Giordano A. Leptin, the brain and energy homeostasis: From an apparently simple to a highly complex neuronal system. Rev Endocr Metab Disord (2022) 23:87–101. doi: 10.1007/s11154-021-09636-2 33822303

[64] Obradovic M Sudar-Milovanovic E Soskic S Essack M Arya S Stewart AJ. Leptin and obesity: role and clinical implication. Front Endocrinol (Lausanne) (2021) 12:585887. doi: 10.3389/fendo.2021.585887 34084149 PMC8167040

[65] Cowley MA Smart JL Rubinstein M Cerdán MG Diano S Horvath TL. Leptin activates anorexigenic POMC neurons through a neural network in the arcuate nucleus. Nature (2001) 411:480–4. doi: 10.1038/35078085 11373681

[66] Cui H López M Rahmouni K. The cellular and molecular bases of leptin and ghrelin resistance in obesity. Nat Rev Endocrinol (2017) 13:338–51. doi: 10.1038/nrendo.2016.222 PMC8904083 28232667

[67] Zhang ZJ Wang XM McAlonan GM. Neural acupuncture unit: A new concept for interpreting effects and mechanisms of acupuncture. Evidence-Based Complementary Altern Med (2012) 2012. doi: 10.1155/2012/429412 PMC3310280 22474503

[68] Jiao Z-T Luo Q. Molecular mechanisms and health benefits of ghrelin: A narrative review. Nutrients (2022) 14:4191. doi: 10.3390/nu14194191 36235843 PMC9572668

[69] Kojima M Hosoda H Date Y Nakazato M Matsuo H Kangawa K. Ghrelin is a growth-hormone-releasing acylated peptide from stomach. Nature (1999) 402:656–60. doi: 10.1038/45230 10604470

[70] Liang F Koya D. Acupuncture: Is it effective for treatment of insulin resistance? Diabetes Obes Metab (2010) 12:555–69. doi: 10.1111/j.1463-1326.2009.01192.x 20590731

[71] Zhao X An X Yang C Sun W Ji H Lian F. The crucial role and mechanism of insulin resistance in metabolic disease. Front Endocrinol (Lausanne) (2023) 14:1149239. doi: 10.3389/fendo.2023.1149239 37056675 PMC10086443

[72] Russo B Menduni M Borboni P Picconi F Frontoni S. Autonomic nervous system in obesity and insulin-resistance-the complex interplay between leptin and central nervous system. Int J Mol Sci (2021) 22:5187. doi: 10.3390/ijms22105187 34068919 PMC8156658

[73] Saad MJA Santos A Prada PO. Linking gut microbiota and inflammation to obesity and insulin resistance. Physiol (Bethesda) (2016) 31:283–93. doi: 10.1152/physiol.00041.2015 27252163

[74] Clamp LD Hume DJ Lambert EV Kroff J. Enhanced insulin sensitivity in successful, long-term weight loss maintainers compared with matched controls with no weight loss history. Nutr Diabetes (2017) 7:e282. doi: 10.1038/nutd.2017.31 28628125 PMC5519190

[75] Kershaw EE Flier JS. Adipose tissue as an endocrine organ. J Clin Endocrinol Metab (2004) 89:2548–56. doi: 10.1210/jc.2004-0395 15181022

[76] Zhang G Sun Q Liu C. Influencing factors of thermogenic adipose tissue activity. Front Physiol (2016) 7:29. doi: 10.3389/fphys.2016.00029 26903879 PMC4742553

[77] Zheng J Dobner A Babygirija R Ludwig K Takahashi T. Effects of repeated restraint stress on gastric motility in rats. Am J Physiol Regul Integr Comp Physiol (2009) 296:R1358–65. doi: 10.1152/ajpregu.90928.2008 19261914

[78] Incollingo Rodriguez AC Epel ES White ML Standen EC Seckl JR Tomiyama AJ. Hypothalamic-pituitary-adrenal axis dysregulation and cortisol activity in obesity: A systematic review. Psychoneuroendocrinology (2015) 62:301–18. doi: 10.1016/j.psyneuen.2015.08.014 26356039

[79] Karaca Z Grossman A Kelestimur F. Investigation of the Hypothalamo-pituitary-adrenal (HPA) axis: a contemporary synthesis. Rev Endocr Metab Disord (2021) 22:179–204. doi: 10.1007/s11154-020-09611-3 33770352

[80] Chao AM Jastreboff AM White MA Grilo CM Sinha R. Stress, cortisol, and other appetite-related hormones: Prospective prediction of 6-month changes in food cravings and weight. Obes (Silver Spring) (2017) 25:713–20. doi: 10.1002/oby.21790 PMC5373497 28349668

[81] van der Valk ES Savas M van Rossum EFC. Stress and obesity: are there more susceptible individuals? Curr Obes Rep (2018) 7:193–203. doi: 10.1007/s13679-018-0306-y 29663153 PMC5958156

[82] Gianotti L Belcastro S D’Agnano S Tassone F. The stress axis in obesity and diabetes mellitus: an update. Endocrines (2021) 2:334–47. doi: 10.3390/endocrines2030031

[83] van der Valk E Abawi O Mohseni M Abdelmoumen A Wester V van der Voorn B. Cross-sectional relation of long-term glucocorticoids in hair with anthropometric measurements and their possible determinants: A systematic review and meta-analysis. Obes Rev (2022) 23:e13376. doi: 10.1111/obr.13376 34811866 PMC9285618

[84] van Rossum EFC. Obesity and cortisol: New perspectives on an old theme. Obes (Silver Spring) (2017) 25:500–1. doi: 10.1002/oby.21774 28229549

[85] Lin J Jiang Y Wang G Meng M Zhu Q Mei H. Associations of short sleep duration with appetite-regulating hormones and adipokines: A systematic review and meta-analysis. Obes Rev (2020) 21:e13051. doi: 10.1111/obr.13051 32537891

[86] Liu S Wang X Zheng Q Gao L Sun Q. Sleep deprivation and central appetite regulation. Nutrients (2022) 14:5196. doi: 10.3390/nu14245196 36558355 PMC9783730

[87] Lee JH Cho J. Sleep and obesity. Sleep Med Clin (2022) 17:111–6. doi: 10.1016/j.jsmc.2021.10.009 35216758

[88] Milaneschi Y Simmons WK van Rossum EFC Penninx BW. Depression and obesity: evidence of shared biological mechanisms. Mol Psychiatry (2019) 24:18–33. doi: 10.1038/s41380-018-0017-5 29453413

[89] Fulton S Décarie-Spain L Fioramonti X Guiard B Nakajima S. The menace of obesity to depression and anxiety prevalence. Trends Endocrinol Metab (2022) 33:18–35. doi: 10.1016/j.tem.2021.10.005 34750064

[90] Esser N Legrand-Poels S Piette J Scheen AJ Paquot N. Inflammation as a link between obesity, metabolic syndrome and type 2 diabetes. Diabetes Res Clin Pract (2014) 105:141–50. doi: 10.1016/j.diabres.2014.04.006 24798950

[91] Fernández-Sánchez A Madrigal-Santillán E Bautista M Esquivel-Soto J Morales-González A Esquivel-Chirino C. Inflammation, oxidative stress, and obesity. Int J Mol Sci (2011) 12:3117–32. doi: 10.3390/ijms12053117 PMC3116179 21686173

[92] Zatterale F Longo M Naderi J Raciti GA Desiderio A Miele C. Chronic adipose tissue inflammation linking obesity to insulin resistance and type 2 diabetes. Front Physiol (2019) 10:1607. doi: 10.3389/fphys.2019.01607 32063863 PMC7000657

[93] Reilly SM Saltiel AR. Adapting to obesity with adipose tissue inflammation. Nat Rev Endocrinol (2017) 13:633–43. doi: 10.1038/nrendo.2017.90 28799554

[94] Wueest S Konrad D. The controversial role of IL-6 in adipose tissue on obesity-induced dysregulation of glucose metabolism. Am J Physiology-Endocrinology Metab (2020) 319:E607–13. doi: 10.1152/ajpendo.00306.2020 32715746

[95] Guillemot-Legris O Muccioli GG. Obesity-induced neuroinflammation: beyond the hypothalamus. Trends Neurosci (2017) 40:237–53. doi: 10.1016/j.tins.2017.02.005 28318543

[96] Jais A Brüning JC. Hypothalamic inflammation in obesity and metabolic disease. J Clin Invest (2017) 127:24–32. doi: 10.1172/JCI88878 28045396 PMC5199695

[97] Kullmann S Abbas Z Machann J Shah NJ Scheffler K Birkenfeld AL. Investigating obesity-associated brain inflammation using quantitative water content mapping. J Neuroendocrinol (2020) 32:e12907. doi: 10.1111/jne.12907 33025697

[98] Larabee CM Neely OC Domingos AI. Obesity: a neuroimmunometabolic perspective. Nat Rev Endocrinol (2020) 16:30–43. doi: 10.1038/s41574-019-0283-6 31776456

[99] Attaye I Warmbrunn MV Boot A van der Wolk SC Hutten BA Daams JG. Gastroenterology. A systematic review and meta-analysis of dietary interventions modulating gut microbiota and cardiometabolic diseases-striving for new standards in microbiome studies. Gastroenterology (2022) 162:1911–32. doi: 10.1053/j.gastro.2022.02.011 35151697

[100] Clemente J Ursell L Wegener Parfrey L Knight R. The impact of the gut microbiota on human health: an integrative view. Cell (2012) 148:1258–70. doi: 10.1016/j.cell.2012.01.035 PMC5050011 22424233

[101] Chow J Lee S Shen Y Khosravi A Mazmanian S. Host–bacterial symbiosis in health and disease. Adv Immunol (2010) 107:243–74. doi: 10.1016/B978-0-12-381300-8.00008-3 PMC3152488 21034976

[102] Kim S Covington A Pamer E. The intestinal microbiota: Antibiotics, colonization resistance, and enteric pathogens. Immunol Rev (2017) 279:90–105. doi: 10.1111/imr.12563 28856737 PMC6026851

[103] Jandhyala SM Talukdar R Subramanyam C Vuyyuru H Sasikala M Nageshwar Reddy D. Role of the normal gut microbiota. World J Gastroenterol (2015) 21:8787–803. doi: 10.3748/wjg.v21.i29.8787 PMC4528021 26269668

[104] Carmody RN Bisanz JE. Roles of the gut microbiome in weight management. Nat Rev Microbiol (2023) 21(8):535–50. doi: 10.1038/s41579-023-00888-0 PMC13306846 37138047

[105] Degruttola A Low D Mizoguchi A Mizoguchi E. Current understanding of dysbiosis in disease in human and animal models. Inflammation Bowel Dis (2016) 22(5):1137–50. doi: 10.1097/MIB.0000000000000750 PMC4838534 27070911

[106] Lozupone CA Stombaugh JI Gordon JI Jansson JK Knight R. Diversity, stability and resilience of the human gut microbiota. Nature (2012) 489:220–30. doi: 10.1038/nature11550 PMC3577372 22972295

[107] Casals-Pascual C González A Vázquez-Baeza Y Song SJ Jiang L Knight R. Microbial diversity in clinical microbiome studies: sample size and statistical power considerations. Gastroenterology (2020) 158(6):1524–28. doi: 10.1053/j.gastro.2019.11.305 31930986

[108] Yu Z Wang Y Yu Z Lu M Xu B. Crosstalk between adipose tissue and the microbiota-gut-brain axis in metabolic diseases. Int J Biol Sci (2022) 18:1706–23. doi: 10.7150/ijbs.68786 PMC8898354 35280695

[109] Bouter KE van Raalte DH Groen AK Nieuwdorp M. Gastroenterology. Role of the gut microbiome in the pathogenesis of obesity and obesity-related metabolic dysfunction. Gastroenterology (2017) 152:1671–8. doi: 10.1053/j.gastro.2016.12.048 28192102

[110] Gershon MD Margolis KG. The gut, its microbiome, and the brain: connections and communications. J Clin Invest (2021) 131(18):e143768. doi: 10.1172/JCI143768 34523615 PMC8439601

[111] Carabotti M Scirocco A Maselli M Severi C. The gut-brain axis: Interactions between enteric microbiota, central and enteric nervous systems. Ann gastroenterology : Q Publ Hellenic Soc Gastroenterol (2015) 28:203–9. PMC4367209 25830558

[112] Bonaz B SinNiger V Pellissier S. Therapeutic potential of vagus nerve stimulation for inflammatory bowel diseases. Front Neurosci (2021) 15:650971. doi: 10.3389/fnins.2021.650971 33828455 PMC8019822

[113] Bonaz B SinNiger V Pellissier S. The vagus nerve in the neuro-immune axis: implications in the pathology of the gastrointestinal tract. Front Immunol (2017) 8:1452. doi: 10.3389/fimmu.2017.01452 29163522 PMC5673632

[114] Breit S Kupferberg A Rogler G Hasler G. Vagus nerve as modulator of the brain–gut axis in psychiatric and inflammatory disorders. Front Psychiatry (2018) 9:44. doi: 10.3389/fpsyt.2018.00044 29593576 PMC5859128

[115] Yu Z. Neuromechanism of acupuncture regulating gastrointestinal motility. World J Gastroenterol (2020) 26:3182–200. doi: 10.3748/wjg.v26.i23.3182 PMC7336328 32684734

[116] Chang YM El-Zaatari M Fau - Kao JY Kao JY Expert Rev Gastroenterol H. Does stress induce bowel dysfunction? Expert Rev Gastroenterol Hepatol (2014) 8:583–5. doi: 10.1586/17474124.2014.911659 PMC4249634 24881644

[117] O’Mahony SM Clarke G Borre YE Dinan TG Cryan JF. Serotonin, tryptophan metabolism and the brain-gut-microbiome axis. Behav Brain Res (2015) 277:32–48. doi: 10.1016/j.bbr.2014.07.027 25078296

[118] Lai Y Liu C-W Yang Y Hsiao Y-C Ru H Lu K. High-coverage metabolomics uncovers microbiota-driven biochemical landscape of interorgan transport and gut-brain communication in mice. Nat Commun (2021) 12:6000. doi: 10.1038/s41467-021-26209-8 34667167 PMC8526691

[119] Torres-Fuentes C Schellekens H Dinan TG Cryan JF Lancet Gastroenterol H. The microbiota-gut-brain axis in obesity. Lancet Gastroenterol Hepatol (2017) 2:747–56. doi: 10.1016/S2468-1253(17)30147-4 28844808

[120] De Vadder F Grasset E Mannerås Holm L Karsenty G Macpherson A Olofsson L. Gut microbiota regulates maturation of the adult enteric nervous system *via* enteric serotonin networks. Proc Natl Acad Sci (2018) 115:201720017. doi: 10.1073/pnas.1720017115 PMC6016808 29866843

[121] Clemmensen C Müller T Woods S Berthoud H-R Seeley R Tschöp M. Gut-brain cross-talk in metabolic control. Cell (2017) 168:758–74. doi: 10.1016/j.cell.2017.01.025 PMC5839146 28235194

[122] van Son JA-O Koekkoek LL La Serlie MJ Nieuwdorp M. The role of the gut microbiota in the gut-brain axis in obesity: mechanisms and future implications. Int J Mol Sci (2021) 22:2993. doi: 10.3390/ijms22062993 33804250 PMC7999163

[123] Voigt J-P Fink H. Serotonin controlling feeding and satiety. Behav Brain Res (2014) 277:14–31. doi: 10.1016/j.bbr.2014.08.065 25217810

[124] van Galen KA ter Horst KW Booij J la Serlie MJ. The role of central dopamine and serotonin in human obesity: lessons learned from molecular neuroimaging studies. Metabolism (2018) 85:325–39. doi: 10.1016/j.metabol.2017.09.007 28970033

[125] Turnbaugh PJ Ley RE Mahowald MA Magrini V Mardis ER Gordon JI. An obesity-associated gut microbiome with increased capacity for energy harvest. Nature (2006) 444:1027–31. doi: 10.1038/nature05414 17183312

[126] Ley R Bäckhed F Turnbaugh P Lozupone C Knight R Gordon J. Obesity alters gut microbial ecology. Proc Natl Acad Sci U.S.A. (2005) 102:11070–5. doi: 10.1073/pnas.0504978102 PMC1176910 16033867

[127] Ridaura V Faith J Rey F Cheng J Duncan A Kau A. Gut microbiota from twins discordant for obesity modulate metabolism in mice. Science (2013) 341:1241214. doi: 10.1126/science.1241214 24009397 PMC3829625

[128] Van Hul M Cani PA-O Nat Rev E. The gut microbiota in obesity and weight management: microbes as friends or foe? Nat Rev Endocrinol (2023) 19:258–71. doi: 10.1038/s41574-022-00794-0 36650295

[129] Proença I Allegretti J Bernardo W Moura D Neto A Matsubayashi C. Fecal microbiota transplantation improves metabolic syndrome parameters: systematic review with meta-analysis based on randomized clinical trials. Nutr Res (2020) 83:1–14. doi: 10.1016/j.nutres.2020.06.018 32987284

[130] Kim S-Y Shin I-S Park Y-J. Comparative effectiveness of a low-calorie diet combined with acupuncture, cognitive behavioral therapy, meal replacements, or exercise for obesity over different intervention periods: A systematic review and network meta-analysis. Front Endocrinol (Lausanne) (2022) 13:772478. doi: 10.3389/fendo.2022.772478 36093081 PMC9458910

[131] Sui Y Zhao HL Wong VCW Brown N Li XL Kwan AKL. A systematic review on use of Chinese medicine and acupuncture for treatment of obesity. Obes Rev (2012) 13:409–30. doi: 10.1111/j.1467-789X.2011.00979.x 22292480

[132] Yin Y Zhao Q Li S Jiang H Yin C Chen H. Efficacy of acupuncture and moxibustion therapy for simple obesity in adults: A meta-analysis of randomized controlled trials. Medicine (2022) 101:e31148. doi: 10.1097/MD.0000000000031148 36316908 PMC9622642

[133] Cui J Song W Jin Y Xu H Fan K Lin D. Research progress on the mechanism of the acupuncture regulating neuro-endocrine-immune network system. Vet Sci (2021) 8(10):149. doi: 10.3390/vetsci8080149 34437474 PMC8402722

[134] World Health Organization. WHO global report on traditional and complementary medicine 2019 (2019). Available at: https://apps.who.int/iris/handle/10665/312342.

[135] Wang M Liu W Ge J Liu S. The immunomodulatory mechanisms for acupuncture practice. Front Immunol (2023) 14:1147718. doi: 10.3389/fimmu.2023.1147718 37090714 PMC10117649

[136] Cho ZH Hwang SC Wong EK Son YD Kang CK Park TS. Neural substrates, experimental evidences and functional hypothesis of acupuncture mechanisms. Acta Neurol Scand (2006) 113:370–7. doi: 10.1111/j.1600-0404.2006.00600.x 16674603

[137] Lacey JM Tershakovec AM Foster GD. Acupuncture for the treatment of obesity: a review of the evidence. Int J Obes Relat Metab Disord (2003) 27:419–27. doi: 10.1038/sj.ijo.0802254 12664074

[138] Darbandi M Darbandi S Owji AA Mokarram P Mobarhan MG Fardaei M. Auricular or body acupuncture: which one is more effective in reducing abdominal fat mass in Iranian men with obesity: a randomized clinical trial. J Diabetes Metab Disord (2014) 13:92. doi: 10.1186/s40200-014-0092-3 25505744 PMC4261582

[139] Li X Jia H-X Yin D-Q Zhang Z-J. Acupuncture for metabolic syndrome: systematic review and meta-analysis. Acupuncture Med (2021) 39:253–63. doi: 10.1177/0964528420960485 33032446

[140] Han X Gao Y Yin X Zhang Z Lao L Chen Q. The mechanism of electroacupuncture for depression on basic research: a systematic review. Chin Med (2021) 16:10. doi: 10.1186/s13020-020-00421-y 33436036 PMC7805231

[141] Zhang B Shi H Cao S Xie L Ren P Wang J. Revealing the magic of acupuncture based on biological mechanisms: A literature review. Biosci Trends (2022) 16:73–90. doi: 10.5582/bst.2022.01039 35153276

[142] White A CM Effective needling techniques FJ. An introduction to western medical acupuncture. Churchill Livingstone (2008). pp. 142–52. doi: 10.1016/B978-0-443-07177-5.00012-X.

[143] Deng H Shen X. The mechanism of moxibustion: ancient theory and modern research. Evidence-Based Complementary Altern Med (2013) 2013:1–7. doi: 10.1155/2013/379291 PMC3789413 24159344

[144] Li F He T Xu Q Lin L-T Li H Liu Y. What is the Acupoint? A preliminary review of Acupoints. Pain Med (2015) 16:1905–15. doi: 10.1111/pme.12761 25975413

[145] Wang L-H Huang W Wei D Ding D-G Liu Y-R Wang J-J. Mechanisms of acupuncture therapy for simple obesity: an evidence-based review of clinical and animal studies on simple obesity. Evid Based Complement Alternat Med (2019) 2019:5796381. doi: 10.1155/2019/5796381 30854010 PMC6378065

[146] Bintoro DA Nareswari I. The role of electroacupuncture in the regulation of appetite-controlling hormone and inflammatory response in obesity. Med Acupunct (2021) 33:264–8. doi: 10.1089/acu.2020.1500 PMC8403175 34471444

[147] Tian N Wang F Tian D-R Zou Y Wang S-W Guan L-L. Electroacupuncture suppresses expression of gastric ghrelin and hypothalamic NPY in chronic food restricted rats. Peptides (NY) (2006) 27:2313–20. doi: 10.1016/j.peptides.2006.03.010 16644064

[148] Wang L Yu C-C Li J Tian Q Du Y-J. Mechanism of action of acupuncture in obesity: A perspective from the hypothalamus. Front Endocrinol (Lausanne) (2021) 12:632324. doi: 10.3389/fendo.2021.632324 33868169 PMC8050351

[149] Wang F Tian DR Tso P Han JS. Arcuate nucleus of hypothalamus is involved in mediating the satiety effect of electroacupuncture in obese rats. Peptides (NY) (2011) 32:2394–9. doi: 10.1016/j.peptides.2011.10.019 22064014

[150] Ji B Hu J Ma S. Effects of electroacupuncture Zusanli (ST36) on food intake and expression of POMC and TRPV1 through afferents-medulla pathway in obese prone rats. Peptides (NY) (2013) 40:188–94. doi: 10.1016/j.peptides.2012.10.009 PMC3646998 23116614

[151] Gong M Wang X Mao Z Shao Q Xiang X Xu B. Effect of electroacupuncture on leptin resistance in rats with diet-induced obesity. Am J Chin Med (Gard City N Y) (2012) 40:511–20. doi: 10.1142/S0192415X12500395 22745067

[152] Johansson J Feng Y Shao R Lönn M Billig H Stener-Victorin E. Intense electroacupuncture norMalizes insulin sensitivity, increases muscle GLUT4 content, and improves lipid profile in a rat model of polycystic ovary syndrome. Am J Physiol Endocrinol Metab (2010) 299:E551–9. doi: 10.1152/ajpendo.00323.2010 20663984

[153] Liang F Chen R Nakagawa A Nishizawa M Tsuda S Wang H. Low-frequency electroacupuncture improves insulin sensitivity in obese diabetic mice through activation of SIRT1/PGC-1α in skeletal muscle. Evid Based Complement Alternat Med (2011) 2011:735297. doi: 10.1155/2011/735297 20981161 PMC2964507

[154] Benrick A Kokosar M Hu M Larsson M Maliqueo M Marcondes RR. Autonomic nervous system activation mediates the increase in whole-body glucose uptake in response to electroacupuncture. FASEB J (2017) 31:3288–97. doi: 10.1096/fj.201601381R 28404742

[155] Lee Y-C Li T-M Tzeng C-Y Chen Y-I Ho W-J Lin J-G. Electroacupuncture at the zusanli (ST-36) acupoint induces a hypoglycemic effect by stimulating the cholinergic nerve in a rat model of streptozotocine-induced insulin-dependent diabetes mellitus. Evid Based Complement Alternat Med (2011) 2011:650263. doi: 10.1093/ecam/neq068 21799686 PMC3136799

[156] Gong M Cao C Chen F Li Q Bi X Sun Y. Electroacupuncture attenuates hepatic lipid accumulation *via* amp-activated protein kinase (Ampk) activation in obese rats. Acupuncture Med (2016) 34:209–14. doi: 10.1136/acupmed-2015-010798 26619891

[157] Cabioğlu MT Ergene N. Electroacupuncture therapy for weight loss reduces serum total cholesterol, triglycerides, and LDL cholesterol levels in obese women. Am J Chin Med (Gard City N Y) (2005) 33:525–33. doi: 10.1142/S0192415X05003132 16173527

[158] Giralt M Villarroya F. White, brown, beige/brite: different adipose cells for different functions? Endocrinology (2013) 154:2992–3000. doi: 10.1210/en.2013-1403 23782940

[159] Farmer SR. Obesity: Be cool, lose weight. Nature (2009) 458:839–40. doi: 10.1038/458839a 19370020

[160] Shen W Wang Y Lu S-F Hong H Fu S He S. Acupuncture promotes white adipose tissue browning by inducing UCP1 expression on DIO mice. BMC Complement Altern Med (2014) 14:501. doi: 10.1186/1472-6882-14-501 25514854 PMC4301852

[161] Wang S-J Zhang J-J Qie L-L. Acupuncture relieves the excessive excitation of hypothalamic-pituitary-adrenal cortex axis function and correlates with the regulatory mechanism of GR, CRH, and ACTHR. Evid Based Complement Alternat Med (2014) 2014:495379. doi: 10.1155/2014/495379 24761151 PMC3972872

[162] Ye Z Zhu L Li X-J Gao H-Y Wang J Wu S-B. PC6 electroacupuncture reduces stress-induced autonomic and neuroendocrine responses in rats. Heliyon (2023) 9:e15291. doi: 10.1016/j.heliyon.2023.e15291 37095918 PMC10121450

[163] Furman D Campisi J Verdin E Carrera-Bastos P Targ S Franceschi C. Chronic inflammation in the etiology of disease across the life span. Nat Med (2019) 25:1822–32. doi: 10.1038/s41591-019-0675-0 PMC7147972 31806905

[164] Li N Guo Y Gong Y Zhang Y Fan W Yao K. The anti-inflammatory actions and mechanisms of acupuncture from acupoint to target organs *via* neuro-immune regulation. J Inflammation Res (2021) 14:7191–224. doi: 10.2147/JIR.S341581 PMC8710088 34992414

[165] Bai H Xu S Wu Q Xu S Sun K Wu J. Clinical events associated with acupuncture intervention for the treatment of chronic inflammation associated disorders. Mediators Inflammation (2020) 2020:2675785. doi: 10.1155/2020/2675785 PMC7336212 32684832

[166] Pan W-X Fan AY Chen S Alemi SF. Acupuncture modulates immunity in sepsis: Toward a science-based protocol. Auton Neurosci (2021) 232:102793. doi: 10.1016/j.autneu.2021.102793 33684727

[167] Wang J Lu S Yang F Guo Y Chen Z Yu N. The role of macrophage polarization and associated mechanisms in regulating the anti-inflammatory action of acupuncture: a literature review and perspectives. Chin Med (2021) 16:56. doi: 10.1186/s13020-021-00466-7 34281592 PMC8287695

[168] Oh J-E Kim S-N. Anti-inflammatory effects of acupuncture at ST36 point: A literature review in animal studies. Front Immunol (2021) 12:813748. doi: 10.3389/fimmu.2021.813748 35095910 PMC8790576

[169] Wen C-K Lee T-Y. Electroacupuncture decreases the leukocyte infiltration to white adipose tissue and attenuates inflammatory response in high fat diet-induced obesity rats. Evid Based Complement Alternat Med (2014) 2014:473978. doi: 10.1155/2014/473978 25202333 PMC4150518

[170] Zhao Y Zhou B Zhang G Xu S Yang J Deng S. The effect of acupuncture on oxidative stress: A systematic review and meta-analysis of animal models. PloS One (2022) 17:e0271098. doi: 10.1371/journal.pone.0271098 36084019 PMC9462787

[171] Yu Y-P Ju W-P Li Z-G Wang D-Z Wang Y-C Xie A-M. Acupuncture inhibits oxidative stress and rotational behavior in 6-hydroxydopamine lesioned rat. Brain Res (2010) 1336:58–65. doi: 10.1016/j.brainres.2010.04.020 20399757

[172] Ma Q. Somato-autonomic reflexes of acupuncture. Med Acupunct (2020) 32:362–6. doi: 10.1089/acu.2020.1488 PMC7755849 33362888

[173] Liu S Wang Z Su Y Qi L Yang W Fu M. A neuroanatomical basis for electroacupuncture to drive the vagal-adrenal axis. Nature (2021) 598:641–5. doi: 10.1038/s41586-021-04001-4 PMC9178665 34646018

[174] Torres-Rosas R Yehia G Peña G Mishra P del Rocio Thompson-Bonilla M Moreno-Eutimio MA. Dopamine mediates vagal modulation of the immune system by electroacupuncture. Nat Med (2014) 20:291–5. doi: 10.1038/nm.3479 PMC3949155 24562381

[175] Lin J-G Kotha P Chen Y-H. Understandings of acupuncture application and mechanisms. Am J Transl Res (2022) 14:1469–81. PMC8991130 35422904

[176] Jang JH Yeom MJ Ahn S Oh JY Ji S Kim TH. Acupuncture inhibits neuroinflammation and gut microbial dysbiosis in a mouse model of Parkinson’s disease. Brain Behav Immun (2020) 89:641–55. doi: 10.1016/j.bbi.2020.08.015 32827699

[177] Hong JA-O Chen JA-O Kan JA-O Liu M Yang DA-O. Effects of acupuncture treatment in reducing sleep disorder and gut microbiota alterations in PCPA-induced insomnia mice. Evid Based Complement Alternat Med (2020). doi: 10.1155/2020/3626120 PMC7647758 33178314

[178] Bao CH Wang CY Li GN Yan YL Wang D Jin XM. Effect of mild moxibustion on intestinal microbiota and NLRP6 inflammasome signaling in rats with post-inflammatory irritable bowel syndrome. World J Gastroenterol (2019) 25:4696–714. doi: 10.3748/wjg.v25.i32.4696 PMC6718040 31528095

[179] Xia X Xie Y Gong Y Zhan M He Y Liang X. Electroacupuncture promoted intestinal defensins and rescued the dysbiotic cecal microbiota of high-fat diet-induced obese mice. Life Sci (2022) 309:120961. doi: 10.1016/j.lfs.2022.120961 36116529

[180] Mengzhu S Yujie Z Yafang S Jing G Tingting Z Yuhang W. Electroacupuncture at Tianshu (ST25) and Zusanli (ST36) alleviates stress-induced irritable bowel syndrome in mice by modulating gut microbiota and corticotropin-releasing factor. J Tradit Chin Med (2022) 42:732–40. doi: 10.19852/j.cnki.jtcm.20220719.001 PMC9924689 36083480

[181] Wang L An J Song S Mei M Li W Ding F. Electroacupuncture preserves intestinal barrier integrity through modulating the gut microbiota in DSS-induced chronic colitis. Life Sci (2020) 261:118473. doi: 10.1016/j.lfs.2020.118473 32971101

[182] Song Y-F Pei L-X Chen L Geng H Yuan M-Q Xu W-L. Electroacupuncture relieves irritable bowel syndrome by regulating IL-18 and gut microbial dysbiosis in a trinitrobenzene sulfonic acid-induced post-inflammatory animal model. Am J Chin Med (Gard City N Y) (2020) 48:77–90. doi: 10.1142/s0192415x20500044 31918565

[183] Liang C Wang KY Gong MR Li Q Yu Z Xu B. Electro-acupuncture at ST37 and ST25 induce different effects on colonic motility *via* the enteric nervous system by affecting excitatory and inhibitory neurons. Neurogastroenterol Motil (2018) 30:e13318. doi: 10.1111/nmo.13318 29488287

[184] Sun J Wu X Meng Y Cheng J Ning H Yongjun P. Electro-acupuncture decreases 5-HT, CGRP and increases NPY in the brain-gut axis in two rat models of Diarrhea-predominant irritable bowel syndrome(D-IBS). BMC Complement Altern Med (2015) 15:340. doi: 10.1186/s12906-015-0863-5 26419631 PMC4589130

[185] Zhao J-M Lu J-H Yin X-J Chen X-K Chen Y-H Tang W-J. Comparison of electroacupuncture and moxibustion on brain-gut function in patients with constipation-predominant irritable bowel syndrome: A randomized controlled trial. Chin J Integr Med (2015) 21(11):855–65. doi: 10.1007/s11655-015-2049-x 25847778

[186] Guo J Chen L Wang Y Song Y Zhao Z Zhao T. Electroacupuncture attenuates post-inflammatory IBS-associated visceral and somatic hypersensitivity and correlates with the regulatory mechanism of epac1–piezo2 axis. Front Endocrinol (Lausanne) (2022) 13:918652. doi: 10.3389/fendo.2022.918652 35865309 PMC9294163

[187] Chu D Cheng P Fau - Xiong H Xiong H Fau - Zhang J Zhang J Fau - Liu S Liu S Fau - Hou X Hou X. Electroacupuncture at ST-36 relieves visceral hypersensitivity and decreases 5-HT(3) receptor level in the colon in chronic visceral hypersensitivity rats. Int J Colorectal Dis (2011) 26:569–74. doi: 10.1007/s00384-010-1087-2 21063714

[188] Kotani N Hashimoto H Fau - Sato Y Sato Y Fau - Sessler DI Sessler Di Fau - Yoshioka H Yoshioka H Fau - Kitayama M Kitayama M Fau - Yasuda T. Preoperative intradermal acupuncture reduces postoperative pain, nausea and vomiting, analgesic requirement, and sympathoadrenal responses. Anesthesiology (2001) 95:349–56. doi: 10.1097/00000542-200108000-00015 11506105

[189] Lee I-S Cheon S Park J-Y. Central and peripheral mechanism of acupuncture analgesia on visceral pain: A systematic review. Evidence-Based Complementary Altern Med (2019) 2019:1–22. doi: 10.1155/2019/1304152 PMC6521529 31186654

[190] Yano JM Yu K Donaldson GP Shastri GG Ann P Ma L. Indigenous bacteria from the gut microbiota regulate host serotonin biosynthesis. Cell (2015) 161:264–76. doi: 10.1016/j.cell.2015.02.047 PMC4393509 25860609

[191] Hartstra AV Schüppel V Imangaliyev S Schrantee A Prodan A Collard D. Infusion of donor feces affects the gut-brain axis in humans with metabolic syndrome. Mol Metab (2020) 42:101076. doi: 10.1016/j.molmet.2020.101076 32916306 PMC7536740

[192] Fang S Wang M Zheng Y Zhou S Ji G. Acupuncture and lifestyle modification treatment for obesity: A meta-analysis. Am J Chin Med (Gard City N Y) (2017) 45:1–16. doi: 10.1142/S0192415X1750015X 28231746

[193] Zhang RQ Jiao T Feng-Ying L Yong-Hong M Li-Xin H Xiao-Li Y. Acupuncture for the treatment of obesity in adults: a systematic review and meta-analysis. Postgrad Med J (2017) 93:743. doi: 10.1136/postgradmedj-2017-134969 28689171

[194] Güçel F Bahar B Demirtas C Mit S Cevik C. Influence of acupuncture on leptin, ghrelin, insulin and cholecystokinin in obese women: a randomised, sham-controlled preliminary trial. Acupuncture medicine : J Br Med Acupuncture Soc (2012) 30:203–7. doi: 10.1136/acupmed-2012-010127 22729015

[195] Belivani M Lundeberg T Cummings M Dimitroula C Belivani N Vasilakos D. Immediate effect of three different electroacupuncture protocols on fasting blood glucose in obese patients: a pilot study. Acupuncture medicine : J Br Med Acupuncture Soc (2015) 33:110–4. doi: 10.1136/acupmed-2014-010662 25522743

[196] Yang X He M Tang Q Wang Z Jin D Wu X. Assessment of anti-inflammatory efficacy of acupuncture in patients with inflammatory bowel disease: A systematic review and meta-analysis. Complement Ther Med (2023) 74:102946. doi: 10.1016/j.ctim.2023.102946 36997007

[197] Mazidi M Abbasi-Parizad P Abdi H Zhao B Rahsepar AA Tavallaie S. The effect of electro-acupuncture on pro-oxidant antioxidant balance values in overweight and obese subjects: a randomized controlled trial study. J Complement Integr Med (2017) 15. doi: 10.1515/jcim-2015-0081 29197218

[198] Zheng Y Jiang X Gao Y Yuan L Wang X Wu S. Microbial profiles of patients with antipsychotic-related constipation treated with electroacupuncture. Front Med (Lausanne) (2021) 8:737713. doi: 10.3389/fmed.2021.737713 34722577 PMC8551555

[199] Zhou J Zhou B Kou X Jian T Chen L Lei X. Effect of summer acupoint application treatment (SAAT) on gut microbiota in healthy Asian adults: A randomized controlled trial. Medicine (2023) 102:e32951. doi: 10.1097/md.0000000000032951 36862868 PMC9981433

[200] Zhang Z Mocanu V Cai C Dang J Slater L Deehan E. Impact of fecal microbiota transplantation on obesity and metabolic syndrome—A systematic review. Nutrients (2019) 11(10):2291. doi: 10.3390/nu11102291 31557953 PMC6835402

[201] Santos-Marcos JA Mora-Ortiz M Tena-Sempere M Lopez-MIranda J Camargo A. Interaction between gut microbiota and sex hormones and their relation to sexual dimorphism in metabolic diseases. Biol Sex Differ (2023) 14:4. doi: 10.1186/s13293-023-00490-2 36750874 PMC9903633

[202] Clifton PM. Dietary treatment for obesity. Nat Clin Pract Gastroenterol Hepatol (2008) 5:672–81. doi: 10.1038/ncpgasthep1283 18852729

[203] Tomiyama AJ Mann T Vinas D Hunger JM Dejager J Taylor SE. Low calorie dieting increases cortisol. Psychosom Med (2010) 72:357–64. doi: 10.1097/PSY.0b013e3181d9523c PMC2895000 20368473

[204] Yang C Hao Z Zhang L-L Guo Q. Efficacy and safety of acupuncture in children: an overview of systematic reviews. Pediatr Res (2015) 78:112–9. doi: 10.1038/pr.2015.91 25950453

[205] Wang X Wang H Guan Y Cai R Shen G. Acupuncture for functional gastrointestinal disorders: A systematic review and meta-analysis. J Gastroenterol Hepatol (2021) 36(11):3015–26. doi: 10.1111/jgh.15645 PMC9292355 34342044

[206] Tremmel M Gerdtham UG Nilsson PM Saha S. Economic burden of obesity: A systematic literature review. Int J Environ Res Public Health (2017) 14:435. doi: 10.3390/ijerph14040435 28422077 PMC5409636

[207] Skonnord T Fetveit A Skjeie H Brekke M Grotle M Klovning A. Cost-effectiveness analysis of acupuncture compared with usual care for acute non-specific low back pain: secondary analysis of a randomised controlled trial. Acupuncture Med (2022) 40:123–32. doi: 10.1177/09645284211055747 PMC8873285 34847780

[208] Kim S-Y Lee H Chae Y Park H-J Lee H. A systematic review of cost-effectiveness analyses alongside randomised controlled trials of acupuncture. Acupuncture Med (2012) 30:273–85. doi: 10.1136/acupmed-2012-010178 23099289

[209] Kim T-H Lee MS Birch S Alraek T. Plausible mechanism of sham acupuncture based on biomarkers: A systematic review of randomized controlled trials. Front Neurosci (2022) 16:834112. doi: 10.3389/fnins.2022.834112 35185461 PMC8850388

[210] Matthews J Villanueva K Yee S Thompson-Lastad A. Working toward integrative health equity: reflections from acupuncture implementation within a pediatric primary care safety-net clinic. Integr Med Rep (2022) 1:20–3. doi: 10.1089/imr.2021.0016 PMC9177051 35692897

